# Tissue Specificity of Human Disease Module

**DOI:** 10.1038/srep35241

**Published:** 2016-10-17

**Authors:** Maksim Kitsak, Amitabh Sharma, Jörg Menche, Emre Guney, Susan Dina Ghiassian, Joseph Loscalzo, Albert-László Barabási

**Affiliations:** 1 Center for Complex Networks Research and Department of Physics, Northeastern University, 110 Forsyth Street, 111 Dana Research Center, Boston, MA 02115, USA; 2 Center for Cancer Systems Biology (CCSB) and Department of Cancer Biology, Dana-Farber Cancer Institute, 450 Brookline Ave., 02215 Boston, USA; 3 Channing Division of Network Medicine, Harvard Medical School, 181 Longwood Avenue, Boston, MA 02115, USA; 4 Center for Network Science, Central European University, Nador u. 9, 1051 Budapest, Hungary; 5 CeMM Research Center for Molecular Medicine of the Austrian Academy of Sciences, Lazarettgasse 14, AKH BT 25.3, A-1090 Vienna, Austria; 6 Institute for Research in Biomedicine, Carrer de Baldiri Reixac, 08028 Barcelona, Spain; 7 DZZOM Inc., 111 Huntington Avenue, Boston, MA 02119, USA; 8 Department of Medicine, Brigham and Womens Hospital, Harvard Medical School, 75 Francis Street, Boston, MA 02115, USA; 9 Department of Theoretical Physics, Budapest University of Technology and Economics, H1111, Budapest, Hungary

## Abstract

Genes carrying mutations associated with genetic diseases are present in all human cells; yet, clinical manifestations of genetic diseases are usually highly tissue-specific. Although some disease genes are expressed only in selected tissues, the expression patterns of disease genes alone cannot explain the observed tissue specificity of human diseases. Here we hypothesize that for a disease to manifest itself in a particular tissue, a whole functional subnetwork of genes (disease module) needs to be expressed in that tissue. Driven by this hypothesis, we conducted a systematic study of the expression patterns of disease genes within the human interactome. We find that genes expressed in a specific tissue tend to be localized in the same neighborhood of the interactome. By contrast, genes expressed in different tissues are segregated in distinct network neighborhoods. Most important, we show that it is the integrity and the completeness of the expression of the disease module that determines disease manifestation in selected tissues. This approach allows us to construct a disease-tissue network that confirms known and predicts unexpected disease-tissue associations.

The rapidly increasing knowledge of the role of genetic variants in human disease raises an important question: Why do pathologic variants that exist in the genome of every organ or tissue affect primarily specific tissues, like asthma affecting the lung or schizophrenia the brain? After all, the mutated genes are present in all cells. The accepted answer holds that genes associated with asthma may be expressed only in the lung and those associated with schizophrenia may show elevated expression only in the brain, eliminating the impact of disease-causing variants in other tissues. This assumption is, however, typically not valid: many disease-associated genes are expressed in multiple tissues, most of which do not show pathophysiological manifestations of the disease or of any functional abnormality^1^,^2^. Consider, for example, Huntington’s disease, a neurodegenerative genetic disorder caused by a mutation of the *HTT* gene (excessive repeats of the trinucleotide *CAG*). As shown in Fig. 1A, we find that *HTT* is significantly expressed in CD34 T cells (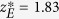), CD56 NK cells (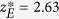), and X721 B lymphoblasts (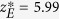), prompting us to ask again, why do the mutations of *HTT* not cause pathophysiological changes in these non-neural tissues?

The driving hypothesis of this paper is that the expression of genes carrying the disease-associated mutation in a particular tissue or organ does not fully explain the tissue-specificity of the disease. Instead, it is the integrity of the cellular subnetwork induced by all disease-associated genes that determines the manifestation of a disease in the tissue. Throughout the text we refer to this cellular subnetwork as a *disease module*^3^,^4^,^5^,^6^,^7^,^8^.

Thus, to understand disease manifestations or pathophenotypes, it is not sufficient to focus narrowly on disease genes and their expression patterns; we must also determine the presence/absence of the disease module, i.e., the tissue-dependent subnetwork, whose breakdown may be responsible for the disease.

This hypothesis is supported by several recent findings. First, disease-associated genes are more likely to exhibit tissue-specific expression than non-disease-associated genes^2^,^9^,^10^,^11^. The integration of gene expression, disease manifestation, molecular network connectivity, and tissue specificity data leads to better predictions of novel disease-gene candidates than any of these elements alone^9^. Similarly, a tissue-specific interactome considerably improves disease gene prioritization compared to prioritization derived from a generic interactome^12^. Our hypothesis is further supported by recent evidence that disease-causing genes tend to have elevated transcript level and increased number of tissues-specific protein interactions in their disease tissue^2^. Similarly, tissue-specific networks were found to accurately predict lineage-specific responses to perturbations, allowing the development of software packages that return the tissue-specific interaction landscape of selected genes^1^.

To test the interplay between the disease module and tissues, we analyze the expression patterns of disease genes and genes in their network vicinity in the human interactome^3^,^4^,^13^,^14^. We show that (i) genes expressed in a specific tissue are localized in the same neighborhood of the interactome, being in each other’s close network-based proximity; (ii) genes expressed in different tissues are segregated in distinct network neighborhoods; and (iii) the integrity of the expression of the disease module determines disease manifestations in selected tissues. These findings offer an accurate and self-consistent formalization of tissue-specificity of human disease modules^15^, offering the graph-theoretical underpinning of relating diseases to specific tissues, consistent with the numerous empirical and experimental studies^1^,^2^,^9^,^10^,^11^,^12^.

## Results

### Tissue-Specific Expression Patterns in the Interactome

In our analysis we use experimentally documented molecular interactions as compiled by Menche *et al.*^15^. The resulting interactome contains 141, 296 physical interactions between 13, 460 proteins, including protein-protein and regulatory interactions, and metabolic pathway and kinase-substrate interactions. To identify the tissue-dependent patterns of expression of each gene, we use global gene expression data for 64 non-diseased tissues from the GNF Atlas^16^. To combine the interactome with the gene expression data we consider molecular interactions only among 10, 434 proteins that were annotated to probe ids (see Supplementary Section II).

Further, we consider a protein-coding gene to be expressed in a tissue if its expression level in this tissue is significantly higher than in other tissues. We use *z*-scores to quantify expression significance and define the significance level at 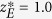 (see Methods and Fig. S1). At this threshold, on average only 10% of genes are expressed in a tissue, ranging from 0.6% for ovary to 44% for X721 B lymphoblasts (Fig. 2A). We also repeated our calculations for a more stringent threshold of 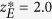, with similar outcomes. We next identified all diseases defined by MeSH that have at least 20 associated genes in the current OMIM and GWAS database^17^,^18^, and then filtered out overly-inclusive diseases, yielding a list of 70 diseases (see Supplementary Section II).

**Figure 1:**
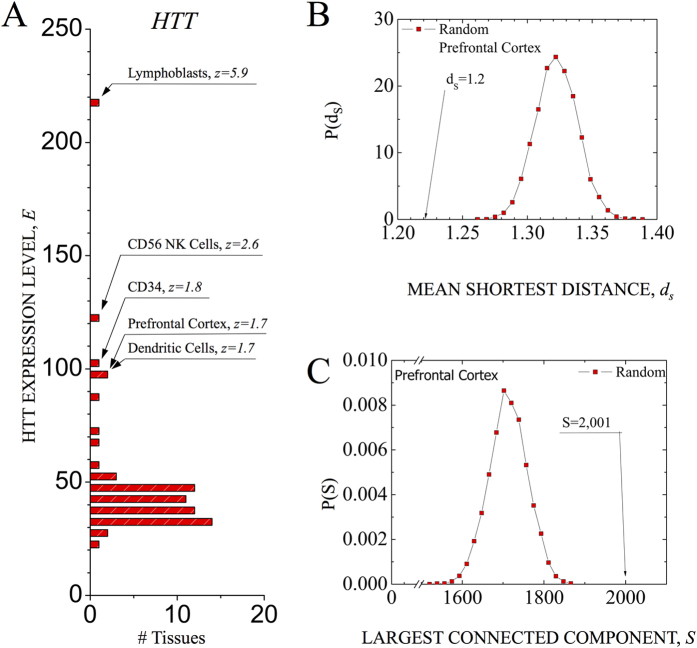
Network-based localization of expressed genes. (**A**) The distribution of expression levels of the *HTT* gene across 64 non-diseased tissues. (**B**) The distribution of the mean shortest distance *d*_*S*_ for randomly chosen sets of genes in the human interactome. The number of randomly selected genes in each set is chosen to match the number of genes expressed in prefrontal cortex at *z*_*E*_ ≥ 1.0. Note that the observed mean shortest distance for genes expressed in prefrontal cortex, *d*_prefr.cort_ = 1.22, is significantly lower than the random expectation. (**C**) The distribution of the connected components size, *P*(*S*), for randomly chosen sets of genes in the human interactome. The number of randomly selected genes in each set is chosen to match the number of genes expressed in prefrontal cortex at *z*_*E*_ ≥ 1.0. Note that the size of the connected component for genes expressed in prefrontal cortex, *S*_prefr.cort_ = 2, 001, is significantly greater than the random expectation.

To illustrate the impact of tissue-specific gene expression patterns on the interactome, consider prefrontal cortex tissue, in which only 2, 644 of the 10, 434 expressed genes are expressed with significance *z*_*E*_ ≥ 1.0. We first asked: are the expressed genes distributed randomly in the interactome or do they tend to agglomerate in some well-defined network neighborhood? We relied on two network-based measures to address this question. (i) We measured the network-based mean shortest distance^15^ among 2, 644 expressed genes, finding 〈*d*_prefr.cort_〉 = 1.22, significantly smaller than the random expectation, 
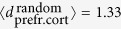
 (*p* value = 8.9 × 10^−17^, *z* score = −11.0) (Fig. 1B). (ii) We next found that 75% of the expressed genes form a single connected subgraph (connected component in graph theory^19^) (*S*_prefr.cort_ = 2, 001, Fig. 1C). This value is significantly greater than the random expectation, 
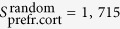
 (*p* value = 8.7 × 10^−10^, *z* score = 6.02) (Fig. 1C) (see Supplementary Section III).

**Figure 2:**
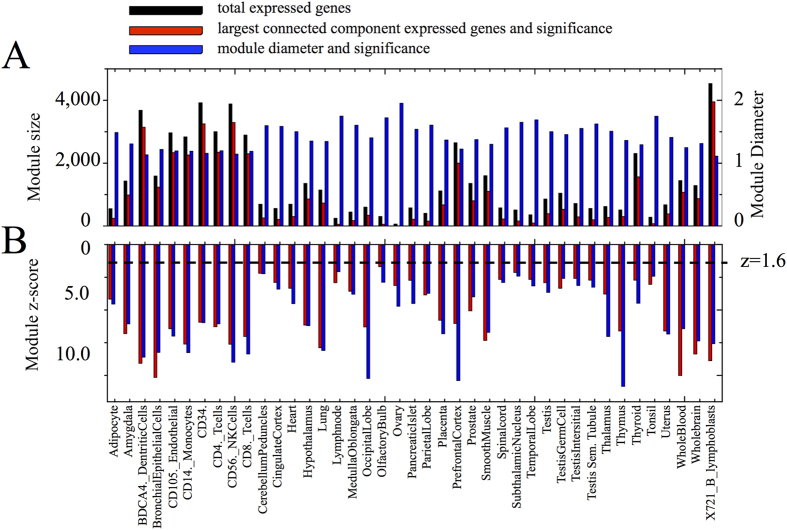
Localization statistics for 41 tissues where a significant fraction of expressed genes forms a connected subgraph. (**A**) The total number of expressed genes (black), the number of genes constituting the largest connected component (module size, red), and the mean shortest distance, *d*_*S*_ (module diameter, blue), calculated for 41 tissues where a significant fraction of expressed genes form a connected subgraph. (**B**) The significance of the observed largest connected components (red) and the significance of the mean shortest distance (blue). The horizontal dashed line corresponds to *z* = 1.6.

Taken together, these results show that the genes expressed in the prefrontal cortex are localized in the same neighborhood of the interactome, and that a very significant fraction of them forms a single connected subgraph. This pattern is not unique to the prefrontal cortex. Indeed, we find that a significant fraction of expressed genes forms a single connected subgraph in 41 of the 64 tissues considered here (see Figs 2, S2 and S3), indicating that each tissue has a characteristic interactome neighborhood in which its proteins agglomerate.

Given that on average only 10% of all genes are expressed in any specific tissue, we next asked: are the genes expressed in different tissues localized in the same or different network neighborhoods? Consider, for example, the hypothalamus and the lung, with *n*_hypothalamus_ = 1, 354 and *n*_lung_ = 1, 141 genes expressed at *z*_*E*_ ≥ 1.0. The number of genes simultaneously expressed in these tissues is *n*_hl_ = 84, significantly lower than the random expectation, 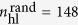 (Jaccard index (see Supplementary Section II), *J*_hl_ = 0.035, 
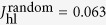
, *p* value = 4.5 × 10^−9^). We also find that the network-based mean shortest distance between gene pairs expressed in the two tissues, *d*_AB_ = 1.30, is significantly larger than random expectation 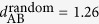 (*p* value = 0.034, *z* score = 1.82) (see Supplementary Section IV). Taken together, these results indicate that the genes expressed in the hypothalamus and the lung are distinct, and that their proteins are located in different interactome neighborhoods.

In contrast, genes expressed in the hypothalamus and prefrontal cortex exhibit both significant overlap and network-based colocalization: *n*_hypothalamus_ = 1, 354 and *n*_prefr.cortex_ = 2, 644, with overlap of *n* = 992, significantly greater than the random expectation, *n*^rand^ = 343 (*J*_hl_ = 0.33, 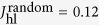, *p* value = 2.4 × 10^−14^). In addition, the mean shortest distance between gene pairs expressed in the two tissues is *d*_AB_ = 0.69, significantly smaller than 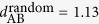 (*p* value < 10^−152^, *z* score = −26.4) (see Supplementary Section II and Fig. S4).

We systematically measured the network-based mean shortest distances *d*_AB_ between gene sets expressed at *z*_*E*_ ≥ 1.0 for all 2, 016 possible tissue pairs. For 1, 415 pairs, the network-based separation is significantly different from the random expectation. Of these, 851 tissue pairs are closer than expected by chance, while 564 pairs show a statistically significant separation. The obtained 1, 415 separations allowed us to build a dendrogram for the hierarchy of tissue clusters (see Fig. S5), predicting three major clusters, one of which consists of several subclusters and contains brain regions, and the remaining contain immune system cells and reproductive tissues.

Taken together, we find that genes expressed in a particular tissue are not scattered randomly throughout the interactome, but tend to form a well-localized connected subnetwork. Subnetworks corresponding to similar tissues tend to overlap, but pathologically distinct tissue pairs tend to agglomerate in different neighborhoods of the interactome. Hence, *we can divide the interactome into tissue-specific neighborhoods*, a partition that has direct implications for our understanding of diseases, as we next demonstrate.

### Disease-Tissue Associations

To illustrate how a disease manifests itself in a particular tissue, consider tauopathies, a class of neurodegenerative diseases associated with the pathological aggregation of *τ* protein within neurons. We find that several genes associated with tauopathies, such as the *MAPT* gene (involved in production of *τ* proteins), are expressed in many tissues, indicating that gene expression alone is a poor predictor of the tissue-specificity of the disease. According to our hypothesis, tauopathies manifest mainly in the brain because the subnetwork of proteins supporting the relevant molecular mechanisms for the disease are expressed integrally only in brain tissue.

**Figure 3:**
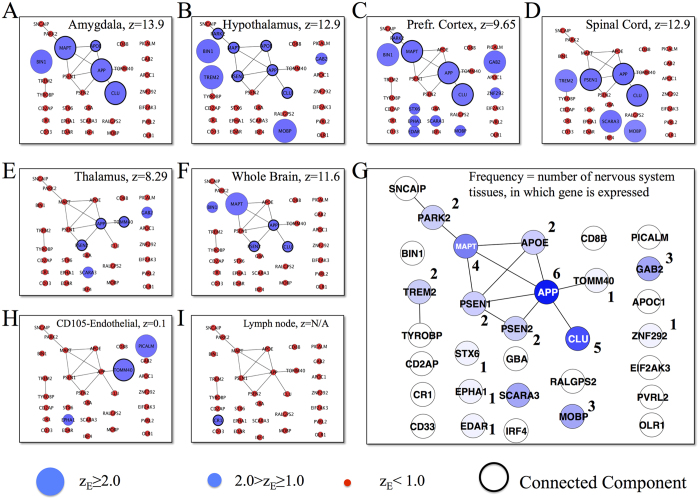
Tissue specificity of tauopathies. (**A**–**F**,**H**,**I**) Expression levels of genes related to tauopathies in (**A**) amygdala, (**B**) hypothalamus, (**C**) prefrontal cortex, (**D**) spinal cord, (**E**) thalamus, (**F**) whole brain, (**H**) CD105-positive endothelial cells, and (**I**) lymph node. Node sizes are chosen to reflect the expression significance of the corresponding genes. Blue nodes correspond to genes with expression significance *z*_*E*_ ≥ 1.0, while red nodes correspond to genes with *z*_*E*_ < 1.0. (**G**), A putative disease modules for tauopathies. The numbers show the number of specific nervous system tissues in which the corresponding gene is expressed.

In support of this hypothesis, we find that the expressed tauopathies genes connect to each other to form a statistically significant connected component only in six tissues, all of which belong to the nervous system (Fig. 3A–F): amygdala, hypothalamus, prefrontal cortex, spinal cord, thalamus, and whole brain. For comparison we also show the subnetwork of tauopathies-related genes in endothelial cells and lymph nodes, where the expressed disease genes do not form a significant connected component, a pattern characteristic of the remaining 60 tissues (Fig. 3H,I).

Combining these observations, we arrive at a putative disease module for tauopathies consisting of nine genes that form a connected subgraph (Fig. 3G). Three of these, *MAPT*, *APP*, and *CLU*, are simultaneously expressed in three of the six brain regions (amygdala, prefrontal cortex, hypothalamus). A key role is played by the amyloid precursor protein *APP*, which not only is the central hub of the module, but also is expressed in all six brain tissues. This network-based conclusion is supported by previous experimental evidence. Indeed, the *APP* on chromosome 21q21.2 is the first causative gene identified for early onset Alzheimer’s disease^20^, a disorder that belongs to the class of tauopathies. Furthermore, *APP* and *MAPT* are known to form soluble complexes that may promote the self aggregation of *APP* into the insoluble forms observed in Alzheimer’s disease^21^. In the nervous system, the expression of *CLU* (clusterin) is elevated in neuropathological conditions, such as Alzheimer’s disease, where *CLU* co-precipitates with *APP*, suggesting a physiological interaction^22^. Finally, recent large genome-wide association studies have identified loci not previously associated with the disease at the *CLU* (also known as *APOJ*) gene (rs11136000, *p* value = 1.4 × 10^−9^)^23^. Since *TOMM*40 is located on chromosome 19, closely adjacent to *APOE*, another gene known to be associated with Alzheimer’s disease^24^, other investigations have suggested that the statistically significant correlation of *TOMM*40 with Alzheimer’s is due to linkage disequilibrium. By contrast, we find *APOE* in Alzheimer’s disease and tauopathies in the connected subgraph and not *TOMM*40, which indicates a greater mechanistic relevance of *APOE* for the disease than *TOMM*40.

**Figure 4:**
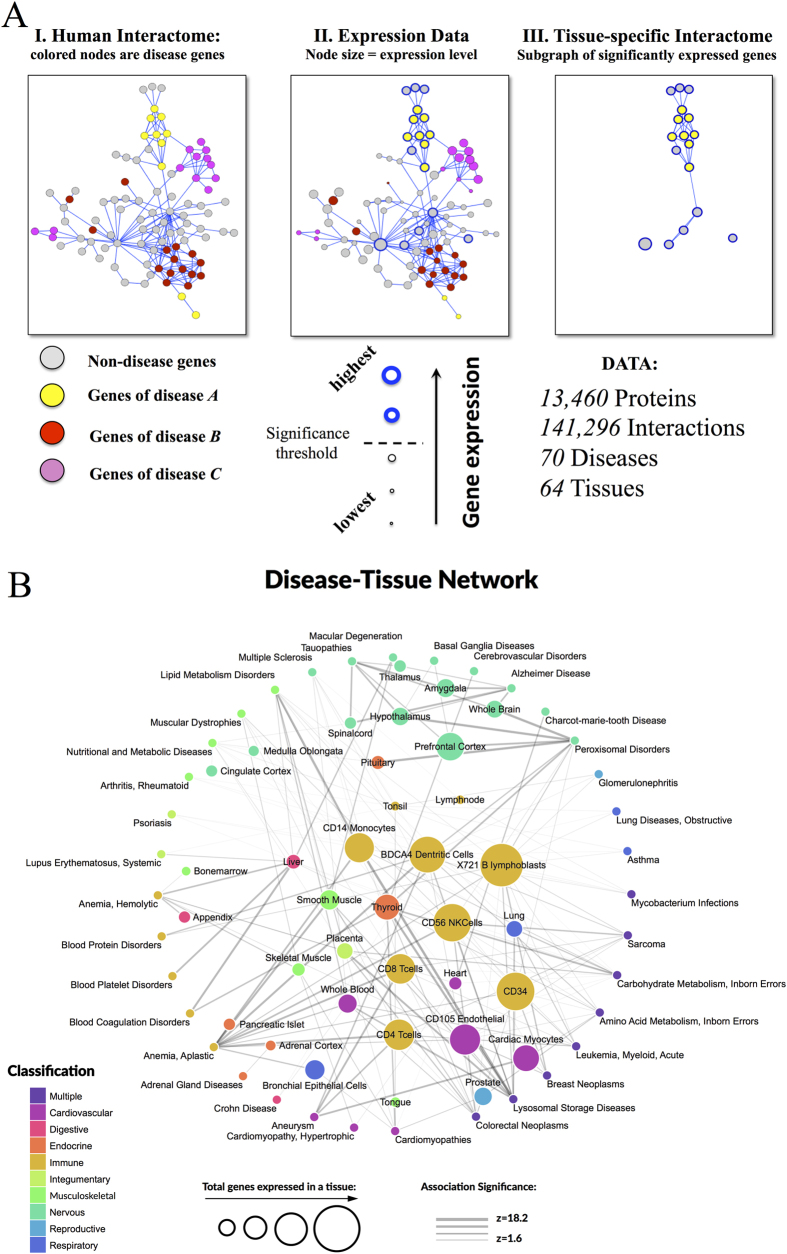
Disease-tissue bipartite network. (**A**) From left to right: I. Human interactome is compiled and disease-related genes are identified. II. Gene expression data are mapped onto the human interactome. The expression level is reflected in the node size. III. A tissue-specific interactome is constructed as a subgraph of the human interactome consisting of genes expressed with significance *z*_*E*_ ≥ 1.0. Disease *A* has significantly connected module within the tissue-specific interactome, while diseases *B* and *C* do not. (**B**) Disease-tissue bipartite network. Tissues are placed within the circle while diseases are positioned along the circumference. Nodes are colored according to tissue classification. The sizes of tissue nodes are proportional to the total number of genes expressed in them. The widths of connecting links correspond to the significance of the association. For higher resolution see Fig. S6.

This example supports our hypothesis that a disease manifests itself only in tissues in which the expressed disease genes form a disease module, i.e., a statistically significant connected subgraph. It also expands earlier findings of elevated expression and protein interaction in specific tissues^1^, indicating that one needs to inspect the integrity of the full disease module to establish the tissue-specificity of a given disease. Building on this hypothesis, we identified all diseases that form a significant subgraph in a particular tissue, following the procedure depicted in Fig. 4A. This strategy allows us to build a disease-tissue bipartite network that links 70 diseases to 64 tissues via 187 links (see Fig. 4B, Supplementary Section V, and Fig. S6). While some diseases manifest in as many as 12 tissues, on average, each of the diseases included in the network manifests in 2.4 tissues. The tissues with the largest number of expressed diseases are BDCA4 dendritic cells (18 diseases), X721 B lymphoblast cells (16 diseases), and CD56 NK cells (12 diseases), appearing as the hubs of the disease-tissue network. For 35 diseases, we did not find a statistically significant module in any tissue, and of the 64 tissues included in this analysis, 29 tissues did not have any disease associated with them. Plausible reasons for these latter two findings are the incompleteness of the interactome^25^,^26^,^27^,^28^ and the limited number of known disease genes.

In addition to a large number of expected disease-tissue associations (e.g., tauopathies and brain tissues), we also find a number of less obvious disease-tissue associations, such as macular degeneration and liver, or lipid metabolism disorders and CD14 monocytes. Next, we discuss several examples to illustrate the predictive power of the developed map and to validate some of the predicted associations.

#### Liver

**Figure 5:**
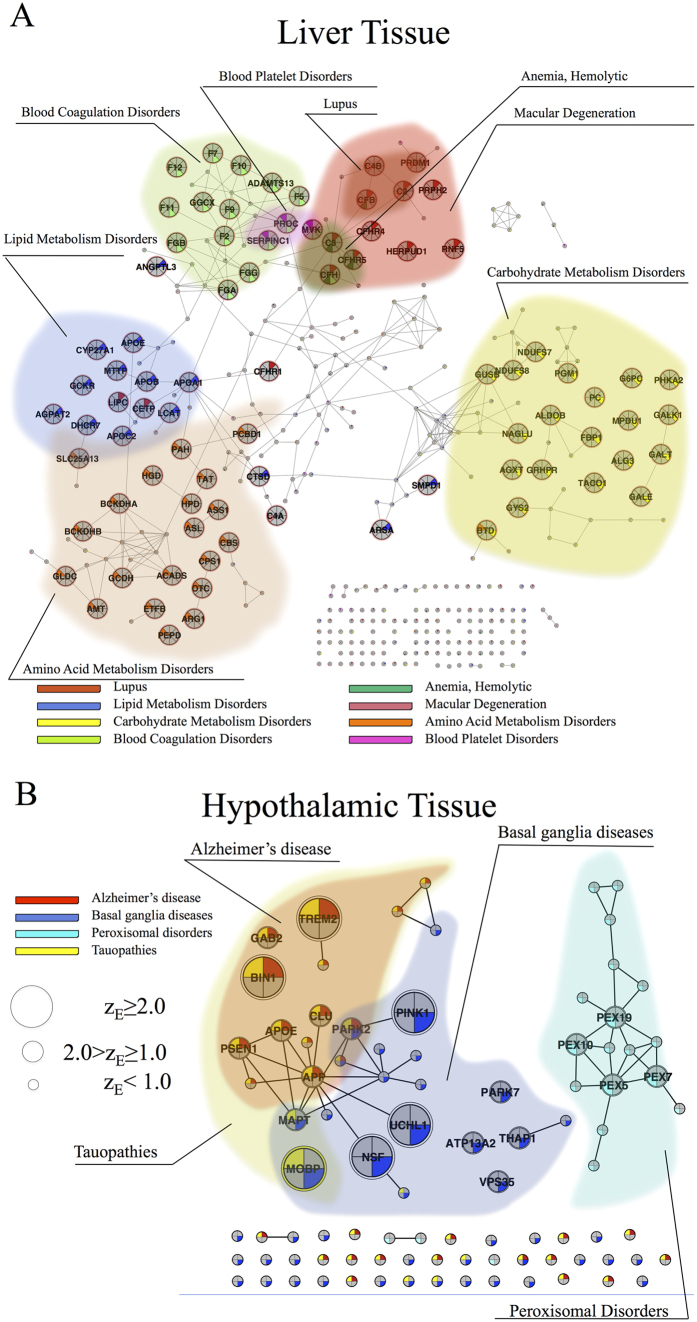
Diseases associated with liver and hypothalamus. (**A**) Diseases associated with the liver-specific interactome. (**B**) Diseases associated with the hypothalamus-specific interactome. Node sizes depend on the expression significance of the corresponding genes. We use pie-chart coloring to reflect disease-gene associations. To improve the readability of diagrams, we grouped genes related to the same disease into modules and highlighted each module accordingly. We excluded disease genes associated with nutritional and metabolic diseases from both panels owing to the large number of associated genes.

Eight diseases have a statistically significant module in liver tissue. Some, like blood coagulation disorders or certain types of anemia, are expected. Indeed, of the 40 genes related to blood coagulation disorders, 14 are expressed in liver and 12 of the expressed genes form a single connected subgraph (*p* value < 10^−53^, *z* score = 15.4) (see Fig. 5A). Five of them, *F*5, *F*7, *F*9, *F*10, and *F*11, are parts of the extrinsic and intrinsic blood clotting pathways. Others, like macular degeneration, are somewhat more surprising. Interestingly, seven genes associated with macular degeneration (*CFH*, *C*3, *C*2, *CFHR*5, *CFB* and *CFHR*4, *CFHR*1) also form a statistically significant module in liver (*p* value < 10^−26^, *z* score = 10.85, see Fig. 5A). Yet, while macular degeneration is an ophthalmologic disorder, there are plausible molecular reasons for this association with the network of expressed genes in liver. Indeed, genes in the complement pathway, including complement factor *H (CFH*), *C*2/*BF*, and *C*3, are known to be associated with age-related macular degeneration (AMD) (see Fig. 5A). The complement system in the blood of mammals comprises more than 30 proteins that are primarily synthesized in the liver and that circulate in their inactive forms. In addition, *CFH*, a major circulating protein, is mainly produced in liver^29^, and livers of patients with AMD are more likely to produce an abnormal form of *CFH*, which is thought to increase inflammation in the eye.

#### Hypothalamus

In the hypothalamus, only three brain-related diseases form significant modules: tauopathies (*S* = 6, *p* value < 10^−37^, *z* score = 12.9), Alzheimer’s disease (*S* = 4, *p* value < 10^−17^, *z* score = 8.6), and basal ganglia diseases (*S* = 3, *p* value = 1.8 × 10^−7^, *z* score = 5.1) (see Fig. 5B). We also find a significant module for peroxisomal disorders (*S* = 4, *p* value < 10^−65^, *z* score = 17.1), a class of conditions caused by defects in peroxisome function. Neurological dysfunction is a prominent feature of most peroxisomal disorders^30^. Peroxisomal disorders are divided into two groups, the peroxisome biogenesis disorders (PBDs) and the peroxisome single-enzyme peroxisome disorders (PSEDs). The connected component of peroxisomal disorders expressed in the hypothalamus consists of *PEX*19, *PEX*10, *PEX*6, and *PEX*7. *PEX*7 encodes peroxisome targeting signal receptor, while *PEX*19 is proposed to be essential for the proper localization and stability of peroxisomal membrane proteins. At the same time, *PEX*6 is required for membrane fusion in an early step of peroxisome biogenesis^31^. While tauopathies and basal ganglia diseases have overlapping modules, peroxisome disorders are separated from them. Moreover, a correlation has been found between the level of peroxisome proliferation in hypothalamus and protection from *APP* (amyloid)-associated neurodegeneration, a process that is linked to Alzheimer’s disease^32^. Current protein-protein interaction maps are estimated to cover 10–15% of all potential interactions^26^. Hence, the isolation of peroxisome disorders from tauopathies and basal ganglia diseases may be a reflection of this limited interactome coverage.

Taken together, the obtained disease-tissue bipartite network supports our hypothesis that disease modules are tissue-specific. To validate this disease-tissue network we examined diseases associated with liver and hypothalamus. While most of the observed associations, including blood coagulation disorders and anemia in liver, Alzheimer disease, Basal ganglia diseases in hypothalamus are well known, we also identified less known disease tissue associations of macular generation in liver, and peroxisomal disorder in hypothalamus. Our finding also provides new routes to analyze tissue-specific disease modules, helping highlight specific disease genes and the molecular mechanisms mediating the pathobiological relationships between diseases.

### Tissue-specific disease modules

Genes associated with the same disease are known to have similar biological characteristics^33^,^34^,^35^. Yet, the precise mechanistic role at the molecular level remains unknown for most disease genes. This prompts us to ask: Could tissue specificity help filter out genes that may have only limited mechanistic relevance to a disease? To answer this question, we start by inspecting the functional annotations of the disease genes according to the three Gene Ontology categories^36^: biological processes (bp), molecular function (mf), and cellular component (cc).

Functional similarity of two genes is defined as the similarity between the sets of their GO annotations^37^. To define functional similarity of two genes we use Simpson and Jaccard similarity coefficients (see Methods). We report the results for the Simpson coefficient in the main text; results obtained with Jaccard similarity are qualitatively similar and are reported in Supplementary Section IID. The functional similarity of a group of genes is then given by the average pairwise functional similarity. For example, for the 40 genes associated with blood coagulation disorders (BCD), the average pairwise biological process similarity is bp(BCD) = 0.36, the average molecular function similarity is mf(BCD) = 0.48, and the average cellular component similarity is cc(BCD) = 0.77 (Supplementary Section IID). Yet, only 14 of the 40 BCD genes are expressed in liver, and these 14 have higher functional similarity values in all three categories: bp(BCD, liver) = 0.44, mf(BCD, liver) = 0.63, and cc(BCD, liver) = 0.91, suggesting that the disease genes excluded by tissue specificity are those whose functional relatedness is smaller.

We, therefore, hypothesize that tissue specificity acts as a “cleansing” or filtering procedure, automatically eliminating the potentially false positive BCD genes (*Hypothesis A*). Alternatively, the observed increase in the functional similarity of disease genes could occur solely due to the fact that tissue-specific genes are functionally more uniform (*Hypothesis B*).

To test the validity of *Hypothesis B* we measured tissue-specific GO similarities for all disease-tissue pairs and calculated their *deviations* from GO similarities measured for all disease genes, Δbp(*d*, *t*) = bp(*d*, *t*) − bp(*d*). Here bp(*d*, *t*) is the biological process similarity of genes associated with disease *d* that are expressed in tissue *t*, and bp(*d*) is the biological process similarity of all genes of disease *d*. We also define Δmf(*d*, *t*) and Δcc(*d*, *t*) in a similar way, using molecular function and cellular component categories, respectively (see Supplementary Section IID). Positive values of Δbp(*d*, *t*) indicate that tissue-specific disease genes are more similar to each other than all genes associated with the disease are similar to each other. For instance, Δbp(BCD, liver) = 0.08, Δmf(BCD, liver) = 0.15, and Δcc(BCD, liver) = 0.14.

**Figure 6:**
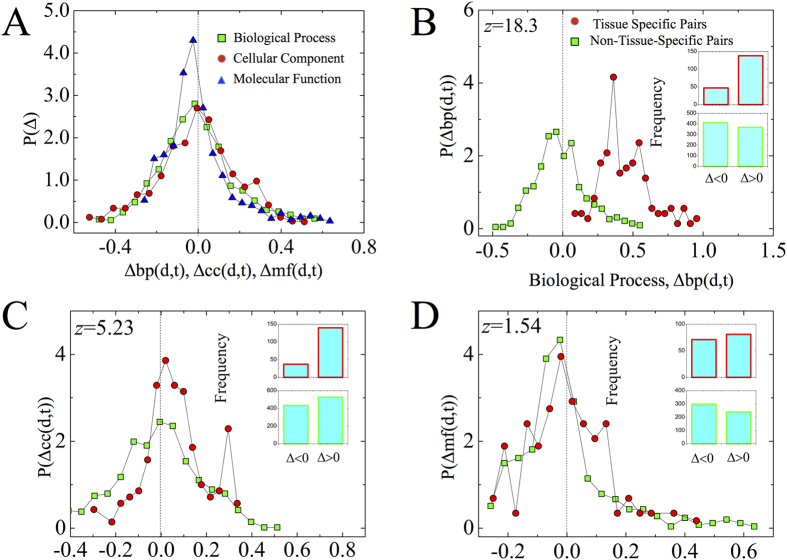
Functional similarity of tissue-specific disease genes. (**A**–**D**), Distributions of changes in GO similarities: (**A**), The distributions of Δbp(*d*, *t*), Δcc(*d*, *t*), and Δmf(*d*, *t*) obtained for all disease-tissue pairs. Measured mean similarity values are, respectively, *M*(Δbp) = 7.6 × 10^−3^, *M*(Δcc) = 0.034, and *M*(Δmf) = −6.3 × 10^−3^. (**B**), Δbp(*d*, *t*), (C), Δcc(*d*, *t*), and (**D**) Δmf(*d*, *t*) for linked disease-tissue pairs (red circles) and non-linked disease-tissue pairs (green squares). The insets in panels (**B**–**D**) compare the total number of Δ > 0 cases with the total number of Δ < 0 cases. Listed *z*-scores are the results of the Mann-Whitney U test applied to the comparison of GO similarities of linked disease-tissue pairs and non-linked disease tissue pairs.

We first calculated the distributions of the deviations Δbp(*d*, *t*), Δmf(*d*, *t*), and Δcc(*d*, *t*) for all possible disease-tissue pairs (Fig. 6A). For all GO categories, we have nearly equal numbers of disease-tissue pairs with positive and negative deviations, which results in median values close to zero (Fig. 6A). This finding indicates that the functional similarity of disease genes Δbp(*d*, *t*) may increase or decrease depending on the disease-tissue pair (*d*, *t*) considered. Note that if hypothesis *B* were true, deviations would be positive for all disease-tissue pairs, including those diseases that have no significant module in the tissue. Hence, the results of Fig. 6A rule out *Hypothesis B*.

To test *Hypothesis A*, the expectation that disease genes are functionally more uniform only in tissues in which they have a significant connected component, we analyzed the distributions of Δbp(*d*, *t*), Δcc(*d*, *t*), and Δmf(*d*, *t*) separately for disease-tissue pairs in which disease genes form a significant connected component (set *CC*) and in which they do not (set *N* − *CC*). Even though the two distributions look visually similar (Fig. 6B–D), the Mann-Whitney U test performed to compare the distributions indicates that they are statistically different (*p*_bp_value < 10^−74^). Of all disease-tissue pairs in *CC*, 70% have Δbp(*d*, *t*) > 0, and 30% have Δbp(*d*, *t*) < 0 (see top inset of Fig. 6B). In the case of *N* − *CC*, we observe 39% of disease-tissue pairs with Δbp(*d*, *t*) > 0 and 61% with Δbp(*d*, *t*) < 0 (bottom inset of Fig. 6B). We, therefore, find that in 70% of tissues the disease genes become functionally more uniform in disease-tissue combinations for which the disease has a significant connected component. We obtained similar results for the cellular component category: (*p*_cc_value = 1.7 × 10^−7^), 79% of disease-tissue pairs have Δcc(*d*, *t*) > 0, while 21% have Δcc(*d*, *t*) < 0 (top and bottom insets of Fig. 6C). In the case of *N* − *CC* we observe that 55% of disease-tissue pairs have Δcc(*d*, *t*) > 0, while 45% have Δcc(*d*, *t*) < 0 (top and bottom insets of Fig. 6C). These results did not hold for the molecular functions GO categories (Fig. 6D): (*p*_mf_value = 0.17). To test the robustness of our results we repeated calculations using the Jaccard coefficient as the similarity measure. Obtained results are qualitatively similar to those obtained with the Simpson coefficient (see Supplementary Section IID).

Taken together, we find that disease genes expressed in a tissue in which a given disease has a significant connected component are functionally more uniform. Consequently, reducing the disease module to a specific tissue acts as a natural filtering procedure, automatically eliminating the functionally and potentially mechanistically less relevant disease genes.

**Figure 7:**
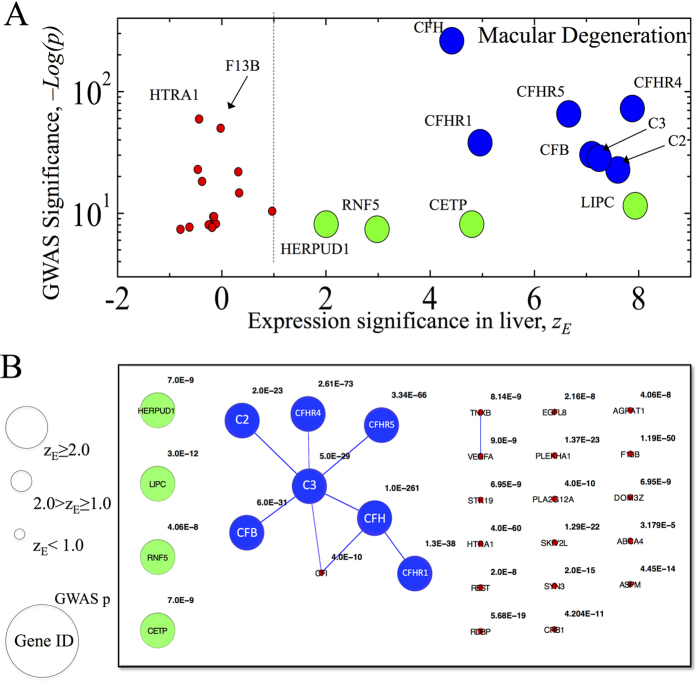
Tissue-specific filtering. To demonstrate the filtering effect of tissue-specificity we consider genes associated with macular degeneration. Node sizes correspond to gene expression significance in liver tissue. Blue nodes correspond to genes in the connected component of the macular degeneration with expression significance of *z*_*E*_ > 1.0. Green nodes correspond to genes with *z*_*E*_ > 1.0 which are not part of the macular degeneration connected component. Red nodes correspond to genes with *z*_*E*_ < 1.0. (**A**), The scatter plot of GWAS association significance as a function of gene expression significance in liver. (**B**), Macular degeneration genes and links among them. Numeric values correspond to GWAS association significance. The most relevant macular degeneration genes form the connected component of the macular degeneration. Note that there are two genes with *z*_*E*_ < 1.0 that have high GWAS significance scores: *HTRA*1 with *p*-value of *p* = 4 × 10^−60^ (−Log(*p*) = 59) and *F*13*B* with *p* = 1.210^−50^ (−Log(*p*) = 50). This observation can be explained as follows. The locus of *p* = 4 × 10^−60^ is close to two the genes *ARMS*2 and *HTRA*1. Genes expressed in the liver are from complementary and lipid metabolism pathways. At the same time, the *ARMS*2 is from inflammatory pathway^38^. *HTRA*1 is not expressed in the liver as per NCBI UniGene. We also note that the apparent association for *CFHR*2-5 and *F*13*B* could be due to their proximity to CFH^39^. This, again, supports our observation that *F*13*B* is not expressed in the liver.

To demonstrate how tissue-specificity can be used to filter out less relevant disease gene associations we consider genes associated with macular degeneration and arthritis. Figure 7A depicts the correlation between the significance of GWAS associations and gene expression levels in liver for macular degeneration. Of the 15 genes expressed at *z*_*E*_ < 1.0, 9 are characterized by relatively low GWAS significance (−Log(*p*) < 6). Of the 11 genes expressed at *z*_*E*_ ≥ 1.0, 7 have GWAS significance of (−Log(*p*) > 20) and 4 have GWAS significance of (7 < −Log(*p*) < 12) (Fig. 7B).

To understand better the liver-specific interactions among the expressed genes we construct the GWAS-based subnetwork of macular degeneration genes (Fig. 7B). We find that 7 genes with GWAS significance −Log(*p*) > 20 (*C*2, *C*3, *CFB*, *CFH*, *CFHR*1, *CFHR*4 and *CFHR*5) form a connected component, while the remaining 4 expressed genes (*LIPC*, *RNF*5, *CETP*, *HERPUD*1) with lower GWAS significance are not connected (Fig. 7B). Taken together, Fig. 7 indicates that the tissue-specific disease module acts as an effective filter, aggregating the diseases genes with high GWAS significance and keeping less relevant genes apart from the disease module.

The analysis of arthritis-related genes expressed in BDCA4 cells is performed in Supplementary Section VI. The examples of macular degeneration and arthritis demonstrate the possibility of using tissue-specificity of disease modules to filter out less relevant disease genes. Yet, at this time, we can not systematically explore tissue-specific filtering effects on all diseases owing to the limited knowledge of GWAS disease-gene associations and the incompleteness of the human interactome.

## Discussion

In this paper we offer a disease module-based approach towards understanding the tissue specificity of human diseases. We showed that for a disease to manifest itself in a particular tissue, a statistically significant functional *subnetwork* of genes associated with the disease needs to be expressed in that tissue. This approach lead to the construction of the disease-tissue network that offers a predictive map of the statistically significant disease-tissue associations. This approach allowed us to examine known disease-tissue relationships and to predict newly definable disease-tissue associations. We also showed that expressed disease genes tend to be more functionally similar if the disease manifests itself in that tissue. These observations can be used as an additional test of the relevance of individual genes to disease.

Throughout this paper, we used gene expression to define tissue-specific interactomes and diseases modules. We considered a gene expressed in a particular tissue when its expression level exceeds the significance threshold of 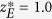. To probe the robustness of our key results, we repeated the analysis for different values of 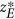 (Supplementary Section V, Figs S4 and S7), finding that:

(i) The localization of disease genes in a particular network-based neighborhood of the human interactome is significant for a wide range of 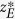 values. The higher 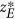, the stronger is the localization, as measured by the mean shortest distance among expressed genes.

(ii) The separations between the subnetworks of expressed genes corresponding to dissimilar tissues increases as 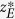 increases.

(iii) A large portion of the identified disease-tissue associations are significant even in the case of a more stringent threshold of 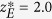. The bipartite disease-tissue network obtained for 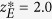 is an almost exact subset of the original network obtained for 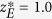 (Supplementary Section V and Fig. S7).

Finally, as we demonstrated in Figs 7 and S8, combining the tissue-specific modules with the interactome allowed us to filter out the less relevant disease genes for arthritis and macular degeneration. Consequently, our findings could considerably improve the predictive power of the interactome for diseases, allowing the construction of more accurate disease modules.

## Methods

### Significance of Gene Expression

We use expression data from 64 non-diseased tissues. We convert probe ids to gene ids using the *U*133 annotation platform. From 13, 460 proteins, only 10, 434 proteins were annotated to probe ids, the basis of our subsequent analysis.

To quantify the expression significance of gene *i* in tissue *t*, we calculate the average expression 〈*E*(*i*)〉 and the standard deviation of a gene’s expression across all considered tissues *σ*_*E*_(*i*). The significance of gene expression in tissue *t* is defined as


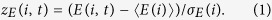


### Tissue Specificity of Disease

To test if disease *d* has a significant connected component in tissue *t*, we first compile the tissue-specific interactome that consists of genes with expression significance *z*_*E*_ ≥ 1.0 in tissue *t* and interactions between them. Next, we map genes associated with disease *d* onto the tissue-specific interactome and then measure the size of the largest connected component *S* and the total number of disease genes *S*_*total*_ expressed in tissue *t* (Fig. 3A).

To test the significance of the observed disease module we assume that disease genes do not preferentially interact in the tissue-specific interactome. With this null hypothesis, we select *S*_*total*_ genes randomly in the tissue-specific interactome and determine the resulting size of the largest connected component *S*^*rand*^. We repeat the same procedure 1, 000 times to obtain the distribution *P*^*rand*^(*S*). Assuming the normality of *P*^*rand*^(*S*), we use the *z*-score to compute the significance of the real data with the threshold of *z* ≥ 1.6 for modules to be larger than expected by chance.

### Gene Ontology Similarity of Disease Modules

The Gene Ontology database^36^ annotates genes and their products with specific molecular functions (mf), biological processes (bf), and cellular components (cc). The molecular function similarity of two genes, *i* and *j*, is defined as the Simpson similarity of their molecular function terms, *S*_*mf*_ (*i*) and *S*_*mf*_ (*j*):


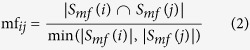


We define the molecular function similarity of a disease *d*, as the average pairwise molecular function similarity of genes related to this disease:


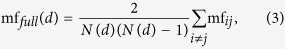


where *N*(*d*) is the number of genes associated with diseases *d* and the summation runs through all pairs of genes associated with disease *d*. Similarly, we define the tissue-specific molecular function similarity of disease *d* as


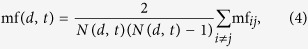


where *N*(*d*, *t*) is the number of genes associated with disease *d* that are expressed in tissue *t*, and the summation runs through all pairs of disease-related genes expressed in tissue *t*. Biological process and cellular component similarities are defined similarly using, respectively, biological process or cellular component annotation terms.

## Additional Information

**How to cite this article**: Kitsak, M. *et al.* Tissue Specificity of Human Disease Module. *Sci. Rep.* **6**, 35241; doi: 10.1038/srep35241 (2016).

## Supplementary Material

Supplementary Information

Supplementary Data File

Supplementary Dataset 1

Supplementary Dataset 2

Supplementary Dataset 3
