# Supplementary material for: Tissue Specificity of Human Disease Module
Source: Sci Rep. 2016 Oct 17;6:35241. doi: 10.1038/srep35241 (PMC5066219; doi:10.1038/srep35241)
Supplement: Supplementary Information [file srep35241-s1.pdf]

# Supplementary Information:
# Tissue Specificity of Human Disease Module

Maksim Kitsak, Amitabh Sharma, Joerg Menche, Emre Guney,
Susan Dina Ghiassian, Joseph Loscalzo, and Albert-László Barabási

## I. INTRODUCTION

This document is organized as follows: In Section II we describe in detail all biological data we use and how we analyze them. In Section III we discuss network-based measures of localization of genes expressed in the same tissue and tools we used to test the significance of localization. In Section IV we discuss network-based measures of separation between genes expressed in different tissues and tools we used to test the significance of these measures. Finally, in Section V we provide details on the construction of the disease-tissue bipartite network.

## II. DATA COMPILATION

### A. Human Interactome

We rely only on experimentally supported pairwise physical protein interactions, excluding interactions extracted from gene expression data or evolutionary considerations. In order to obtain an interactome as complete as currently feasible, we combine several databases:

- Regulatory interactions obtained from the TRANSFAC database [1]. Here nodes represent transcription factors, and connections represent physical binding to regulatory elements. The network consists of 271 transcription factors and 564 regulated genes.
- Binary interactions: We combine several yeast-to-hybrid high-throughput datasets with binary interactions [2–6] from IntAct [7] and MINT [8] datasets. The resulting network consists of $8,120$ proteins and $28,653$ interactions between them.
- Interactions manually curated from literature. We use InAct [7] and MINT [8], BioGRID [9], and HPRD [10] databases, resulting altogether in $11,798$ proteins and $88,349$ interactions among them.
- Metabolic enzyme-coupled interactions: Two enzymes are coupled if they share adjacent reactions in the KEGG and BIGG databases. In total, we have $5,325$ metabolic links between 921 enzymes from Ref. [11].
- Protein complexes: Protein complexes are single molecular units integrating multiple gene products. We use the CORUM database [12], which is a collection of mammalian complexes derived from a variety of experimental tools, from co-immunoprecipitation to co-sedimentation and ion exchange chromatography. The CORUM database yields $2,837$ complexes involving $2,069$ proteins connected by $31,276$ links.
- Kinase-substrate pairs: Protein kinases are important regulators in different biological processes, such as signal transduction. PhosphositePlus [13] provides a network of peptides that can be bound by kinases, yielding in total $6,066$ interactions between $1,843$ proteins.

The union of all interactions yields a network of $13,460$ proteins that are connected by $141,296$ physical interactions. Human Interactome data is included into a supplementary dataset.

### B. Disease-Gene Associations

We integrate two sources of disease-gene annotation:

OMIM: The OMIM database (Online Mendelian Inheritance in Man `http://www.ncbi.nlm.nih.gov/omim` [14] is a comprehensive collection covering all known diseases with a genetic component. We use data compiled by Mottaz *et al* [15] that also includes associations from UniProtKB/Swiss-Prot.

GWAS: The disease-gene associations from Genome-Wide Association Studies (GWAS) are obtained from the PheGenI database (Phenotype-Genotype Integrator; `http://www.ncbi.nlm.nih.gov/gap/PheGenI` [16] that integrates

various NCBI genomic databases. We use a genome-wide significance cutoff of p-value $p \leqslant 5^{-8}$. Disease-gene associations are included into a supplementary dataset.

### C. Gene Expression Data

We use tissue specific gene expression data from Ref. [17]. In our analysis we consider 64 healthy tissues, excluding cancerous and fetal tissues. We use "tissue" here and the main text to refer to specific tissue or organ or cell type from which there are available expression data. We convert probe ids to gene ids using the U133 annotation platform. From 13,460 proteins, only 10,434 proteins were annotated to probe ids, the basis of our subsequent analysis.

To quantify the expression significance of gene $i$ in tissue $t$, we calculate the average expression $\langle E(i) \rangle$ and the standard deviation of a gene's expression across all considered tissues $\sigma_E(i)$. The significance of gene expression in tissue $t$ is defined as

$$z_E(i,t) = (E(i,t) - \langle E(i) \rangle) / \sigma_E(i). \tag{1}$$

We consider gene $i$ expressed in tissue $t$ if its expression significance $z_E(i,t) > 1.0$ (Fig. S1B). We note that under this condition all 10,434 genes used in our analysis are significantly expressed in at least one tissue. While, on average each gene is expressed in 6.5 tissues, we find that some genes can be simultaneously expressed in up to 16 tissues (Fig. S1C). To construct a tissue-specific interactome we include all expressed genes and interactions between them. In doing so we treat each gene equally, regardless of its expression significance and the number of other tissues it is expressed in.

It is important to note that some genes, are simultaneously expressed in similar tissues while other genes may be simultaneously expressed in tissues of different types. For instance, we find that *MAPT* gene is expressed mostly in nervous tissues: BDCA4 Dentritic Cells ($z_E = 3.25$), Cerebellum Peduncles ($z_E = 1.51$), Cingulate Cortex ($z_E = 2.73$), Globus Pallidus ($z_E = 1.18$), Hypothalamus ($z_E = 1.20$), Medulla Oblongata ($z_E = 1.59$), Occipital Lobe ($z_E = 2.25$), Parietal Lobe ($z_E = 1.44$), Prefrontal Cortex ($z_E = 2.73$), Subthalamic Nucleus ($z_E = 1.34$) and Temporal Lobe ($z_E = 1.11$). In contrast, *CLU* gene is expressed in tissues of different types: Amygdala ($z_E = 2.34$), Hypothalamus ($z_E = 1.79$), Liver ($z_E = 1.95$), Olfactory Bulb ($z_E = 1.06$), Placenta ($z_E = 3.39$), Spinal cord ($z_E = 2.91$), and Testis Germ Cell ($z_E = 2.74$). Consistent with these observations, *CLU* is known to participate in several basic biological events such as cell death, tumor progression, and neurodegenerative disorders, while *MAPT* is primarily responsible for the assembly and stability of microtubules. Thus, our observations suggest that tissue-specificity of individual genes could help one decipher their roles in different tissues.

### D. Gene Ontology

GO annotations [18] for all genes are extracted from [`http://www.geneontology.org/`, downloaded Nov. 2011]. We only use high confidence annotations associated with the evidence codes `EXP, IDA, IMP, IGI, IEP, ISS, ISA, ISM,` or `ISO`. In particular, we exclude annotations inferred from physical interactions (evidence code `IPI`) in order to avoid circularity in the evaluation of the GO-based similarity of proteins within the interactome. Following the curation process from [19], we further remove all the associations that have a non-empty "qualifier" column. The original GO files only contain the most specific annotations explicitly. In a last step, we, therefore, add all implicit, more general annotations by up-propagating the given annotations along the full GO tree. Note that the strict filtering procedure reduces the total number of GO terms considerably, such that in the end we use only $\sim 50\%$ of all available terms for *biological processes* (bp) and *molecular function* (mf) and 25% for *cellular component* (cc).

Functional similarity of two genes can be defined as a similarity between the two sets of their Gene Ontology annotations using any similarity index, e.g., the Jacccard index. In this work perform the analysis using independently the Simpson's and the Jaccard indices arriving at qualitatively similar results. Here we report results obtained with the Jaccard index, while results for the Simpson index are reported in the main text.

We quantify the biological process similarity of a given pair of genes $i$ and $j$ by the Jaccard index of their respective biological process annotations $S_{bp}(i)$ and $S_{bp}(j)$:

$$\mathrm{bp}_{ij} = \frac{|S_{bp}(i) \cap S_{bp}(j)|}{|S_{bp}(i) \cup S_{bp}(j)|}. \tag{2}$$

The values of $\mathrm{bp}_{ij}$ range from $\mathrm{bp}_{ij} = 0$ for no shared biological process GO terms, to $\mathrm{bp}_{ij} = 1$ if $i$ and $j$ have identical sets of biological process GO annotations. Molecular function and cellular component similarities are quantified in a

similar way:

$$\mathrm{mf}_{ij} = \frac{|S_{mf}(i) \cap S_{mf}(j)|}{|S_{mf}(i) \cup S_{mf}(j)|}, \tag{3}$$

$$\mathrm{cc}_{ij} = \frac{|S_{cc}(i) \cap S_{cc}(j)|}{|S_{cc}(i) \cup S_{cc}(j)|}, \tag{4}$$

where $S_{mf}(i)$ and $S_{cc}(i)$ are molecular function and biological process annotations of gene $i$.

The overall biological process similarity of a given set of genes is measured by averaging $\mathrm{bp}_{ij}$ over all possible pairs of genes within the set. In particular, we quantify the biological process similarity of genes associated with disease $d$ as:

$$\mathrm{bp}_{full}(d) = \frac{2}{N(d)\,(N(d)-1)} \sum_{i \neq j} \mathrm{bp}_{ij}, \tag{5}$$

where $N(d)$ is the total number of genes associated with disease $d$. Molecular function and cellular component similarities of genes associated with disease $d$ are defined similarly:

$$\mathrm{mf}_{full}(d) = \frac{2}{N(d)\,(N(d)-1)} \sum_{i \neq j} \mathrm{mf}_{ij}, \tag{6}$$

$$\mathrm{cc}_{full}(d) = \frac{2}{N(d)\,(N(d)-1)} \sum_{i \neq j} \mathrm{cc}_{ij}. \tag{7}$$

We also quantify tissue-specific biological process, molecular function and cellular component similarities of disease $d$ by averaging over pairs of disease genes, which are significantly expressed in tissue $t$:

$$\mathrm{bp}(d,t) = \frac{2}{N(d,t)\,(N(d,t)-1)} \sum_{i \neq j} \mathrm{bp}_{ij}, \tag{8}$$

$$\mathrm{mf}(d,t) = \frac{2}{N(d,t)\,(N(d,t)-1)} \sum_{i \neq j} \mathrm{mf}_{ij}, \tag{9}$$

$$\mathrm{cc}(d,t) = \frac{2}{N(d,t)\,(N(d,t)-1)} \sum_{i \neq j} \mathrm{cc}_{ij}, \tag{10}$$

where $N(d,t)$ is the total number of disease genes significantly expressed in tissue $t$.

We measured tissue-specific GO similarities for all disease-tissue pairs and calculated their *deviations* from GO similarities measured for all disease genes,

$$\Delta\mathrm{bp}(d,t) = \mathrm{bp}(d,t) - \mathrm{bp}(d), \tag{11}$$

$$\Delta\mathrm{mf}(d,t) = \mathrm{mf}(d,t) - \mathrm{mf}(d), \tag{12}$$

$$\Delta\mathrm{cc}(d,t) = \mathrm{cc}(d,t) - \mathrm{cc}(d) \tag{13}$$

Positive values of $\Delta\mathrm{bp}(d,t)$ indicate that tissue-specific disease genes are more similar to each other than all genes associated with the disease are similar to each other. We argue using Simpson similarity index that disease genes expressed in a tissue in which a given disease has a significant connected component are functionally more uniform.

To test the robustness of our result we repeated measurements using the Jaccard similarity index, arriving at similar results. We analyzed the distributions of $\Delta\mathrm{bp}(d,t)$, $\Delta\mathrm{cc}(d,t)$, and $\Delta\mathrm{mf}(d,t)$ separately for disease-tissue pairs in which disease genes form a significant connected component (set $CC$) and in which they do not (set $N-CC$). In case of set $CC$, 67% disease-tissue pairs have $\Delta\mathrm{bp}(d,t) > 0$ and 33% have $\Delta\mathrm{bp(d,t)} < 0$. In the case of $N-CC$, we observe 47% of disease-tissue pairs with $\Delta\mathrm{bp}(d,t) > 0$ and 53% with $\Delta\mathrm{bp}(d,t) < 0$. For the cellular component category 63% $\Delta\mathrm{cc}(d,t) > 0$, while 37% have $\Delta\mathrm{cc}(d,t) < 0$ (set $CC$). In the case of $N-CC$ 48% of disease-tissue pairs have $\Delta\mathrm{cc}(d,t) > 0$, while 52% have $\Delta\mathrm{cc}(d,t) < 0$. Similar to the case of Simpson index, our results did not hold for the molecular functions GO categories. These results did not hold for the molecular functions GO categories.

## III. THE LOCALIZATION OF EXPRESSED GENES

We use two complementary measures to quantify the network localization of given set of genes A: (i). The first measure is the size of the largest connected component $S_{\mathrm{A}}$, i.e., the highest number of genes within A that are

directly connected to one another. Since $S_{\mathrm{A}}$ is relatively sensitive to data incompleteness – in extreme cases a single missing link in the interactome may destroy the connected component – we complement our analysis by measuring the distribution of (ii) shortest distances $d_s$ within the gene set: For each of the $N_A$ proteins we determine the *shortest* distance $d_s$ to the next closest protein associated with the same disease, resulting in a distribution $P(d_s)$ of $N_A(N_A-1)/2$ data points. The average value $\langle d_s \rangle$ can be interpreted as the diameter of the gene set on the interactome. There are several possible variations and extensions of this distance measure. In particular, we have explored using *all* pairwise distances instead of only the distance to the next closest protein. We find that while the general results do not depend on the exact choice, $d_s$ is the most predictive quantity, offering higher effect size with statistical significance.

Are the expressed genes distributed randomly in the interactome or do they agglomerate in some well-defined network neighborhood? To answer this question, we rely on two network-based measures. We discuss these measures using the example of prefrontal cortex tissue.

*Mean shortest distance:*

Of the $10,434$ genes considered in our analysis, only $2,644$ are expressed with significance $z_E \geqslant 1.0$ in prefrontal cortex. We first calculate the mean shortest distance among the $2,644$ expressed genes in the human interactome: $d_{prefr.cort} = 1.22$. To assess the significance of the measured mean shortest distance, we randomize the set of expressed genes. Assuming the null hypothesis that expressed genes have no tendency to agglomerate in any particular neighborhood of the interactome, we choose $2,644$ genes at random and determine the mean shortest distance. We repeat the procedure $1,000$ times and construct the resulting distribution $P(d_S^{rand})$ of mean shortest distances among genes chosen at random. As seen on Fig. S2A, $P(d_S^{rand})$ is sharply peaked at $\langle d_S \rangle = 1.33$. We then calculate the z-score for $d_{prefr.cort}$ as:

$$z = \frac{d_{prefr.cort} - \langle d_S \rangle}{\sigma_{d_S^{rand}}}, \tag{14}$$

where $\sigma_{d_S}$ is the standard deviation of $P^{rand}(d_S)$. Assuming the normality of $P^{rand}(d_S)$, we can analytically calculate a corresponding $p$-value for each $z$-score, yielding a threshold of $z \leqslant -1.65$ for $d_S$ to be smaller than expected by chance with $p \leqslant 0.05$. We obtain $z = -11.0$ for prefrontal cortex tissue, indicating the genes expressed in the tissue are significantly closer to each other then expected by chance.

*Size of the largest connected component:*

We consider the subnetwork of $2,644$ genes expressed in the prefrontal cortex tissue and interactions between them. We find that $S_{prefr.cort} = 2,001$ of the expressed genes form the largest connected component, which accounts for 75% genes in the expressed subnetwork. To assess the significance of the measured $S_{prefr.cort}$, we assume the null hypothesis that expressed genes are scattered at random.

We create the subnetwork of $2,644$ randomly selected genes and interactions between them. For the given random subnetwork we calculate the size of its largest connected component $S^{rand}_{prefr.cort}$. We then repeat the same procedure $1,000$ times to obtain distribution $P(S^{rand}_{prefr.cort})$ values (Fig. S2B). We measure the significance of observed $S_{prefr.cort} = 2,001$ by calculating the $z$-score as:

$$z = \frac{S_{prefr.cort} - \langle S^{rand}_{prefr.cort} \rangle}{\sigma_{S^{rand}_{prefr.cort}}}. \tag{15}$$

Assuming the normality of $P(S^{random}_{prefr.cort})$, we use the threshold of $z \geqslant 1.65$ for the modules to be larger than expected by chance with significance of $p \leqslant 0.05$. We obtain $z = 6.02$ in the case of prefrontal cortex. For visualization purposes, we depict the layout of the prefrontal cortex subnetwork (Fig. S2E) and the layout of one of the random subnetworks of the same size (Fig. S2F).

We stress that the observed agglomeration of genes expressed in prefrontal cortex tissue is not specific to the choice of the expression significance threshold $z_E^*$. We repeat our calculations by varying the fraction of the most expressed genes from $f = 10^{-3}$ to $f = 1$ (Fig. S2C and Fig. S2D). This corresponds to varying the expression significance from $z_E^* = 5.22$ to $z_E^* = -1.29$. We observe that the observed agglomeration of genes expressed in the prefrontal cortex is significant for $f \leqslant 0.3$.

We repeat the same analysis of the agglomeration of expressed genes for all 64 tissues and find significant agglomeration in 41 of the considered 64 tissues. Our findings are summarized in Fig. S3A,B.

## IV. GENES EXPRESSED IN DIFFERENT TISSUES

### A. Measures of Separation

We use two complementary measures to quantify the separation between two given sets of genes $A$ and $B$.

The first measure is the Jaccard similarity of the two sets:

$$J_{AB} = \frac{|A \bigcap B|}{|A \bigcup B|}, \tag{16}$$

where $|X|$ corresponds to the number of elements in set $X$. To estimate the significance of the above observation we assume the null hypothesis that the two sets of genes are selected at random. The probability of a particular gene expressed in set $A$ is then $p_A = \frac{|A|}{N}$, where $N$ is the total number of genes considered in the analysis. Similarly, the probability that a particular gene is expressed in set $B$ is given by $p_B = \frac{|B|}{N}$. Assuming that genes are chosen independently for sets $A$ and $B$, the probability that a particular gene is expressed simultaneously in both sets is $p = p_A p_B$. The probability of observing $m$ genes expressed simultaneously in both sets, therefore, follows the binomial distribution $B(N, p)$:

$$P(m; N, p) = C_m^N p^m (1-p)^{N-m}, \tag{17}$$

where $C_m^N$ is the binomial coefficient. Thus, the expected number of genes contained simultaneously in both sets is given by:

$$\langle |A \bigcap B| \rangle \equiv \langle m \rangle = Np = \frac{n_A \times n_B}{N} \tag{18}$$

The significance of observing $|A \bigcap B|$ genes can then be calculated as

$$p = \sum_{m=0}^{|A \bigcap B|} P(m). \tag{19}$$

The second separation measure of two gene sets $A$ and $B$ is the mean shortest pairwise network-based distance $\langle d_{\mathrm{AB}} \rangle$ *between* the genes of the two sets [20]. $\langle d_{\mathrm{AB}} \rangle$ is calculated as follows: for every gene $i$ in set $A$, we determine the *shortest* network based distance $d_i(B)$ to the closest gene from set $B$. The same calculation is then repeated for all genes constituting set $B$: for every gene $j$ in set $B$, we determine the *shortest* network based distance $d_j(A)$ to the closest gene from set $A$. The mean shortest pairwise network-based distance $\langle d_{\mathrm{AB}} \rangle$ is then calculated as

$$\langle d_{\mathrm{AB}} \rangle = \frac{1}{|A| + |B|} \left( \sum_i d_i(B) + \sum_j d_j(B) \right). \tag{20}$$

### B. Separation between genes expressed in different tissues

Are the genes expressed in different tissues localized in the same or in different network neighborhoods?

To answer this question, we first consider hypothalamus and lung tissues. There are $n_{hypothalamus} = 1,354$ and $n_{lung} = 1,141$ genes expressed at $z_E \geqslant 1.0$ in the hypothalamus and the lung, respectively. The number of genes simultaneously expressed in these tissues is $n_{hl} = 84$. The number of genes common to both tissues expected by chance is $\langle n_{hl} \rangle = 148$, which is significantly large then the observed $n_{hl} = 84$ ($p = 4.47 \times 10^{-9}$) We calculate the mean shortest network-based separation between genes expressed in the hypothalamus and lung to be $d_{AB} = 1.30$. Assuming the same null hypothesis, we calculate $d_{AB}^{rand}$ for the two sets of nodes of sizes $n_{hypothalamus}$ and $n_{lung}$ chosen at random. Repeating the same procedure $1,000$ times, we obtain the $P(d_{AB}^{rand})$, which is approximately normal with $\langle d_{AB}^{rand} \rangle = 1.26$ and $\sigma\left(d_{AB}^{rand}\right) = 0.022$. This allows us to use the $z$-score to evaluate the significance of the observed $d_{AB}$

$$z = \frac{d_{AB} - \langle d_{AB}^{rand} \rangle}{\sigma\left(d_{AB}^{rand}\right)} = 1.82 \tag{21}$$

These results indicate that the genes expressed in the hypothalamus and lung are distinct and also located in different network neighborhoods.

We note, however, that other tissue pairs can be closer to each other then expected by chance. For example, genes expressed in hypothalamus and prefrontal cortex, both nervous system tissues, exhibit both significant overlap and network-based colocalization. Indeed, we have $n_{hypothalamus} = 1,354$ and $n_{prefr.cortex} = 2,644$ for genes expressed at $z_E \geqslant 1.0$. The number of genes expressed simultaneously in both tissues is $n_{hp} = 992$, significantly higher than the random expectation, $n_{hp}^{rand} = 343$, ($p = 2.4 \times 10^{-14}$). The mean shortest network-based separation between pairs of genes expressed in the two tissues is $d_{AB} = 0.69$, significantly smaller than $d_{AB}^{random} = 1.12$ ($z = -31.4$).

Our findings are largely independent of the choice of expression significance threshold $z_E^*$. Figure S4 depicts the Jaccard index and the $d_{AB}$ separation for the sets of genes expressed in two tissues as a function of $z_E^*$ and indicates that genes expressed in the two tissues are significantly different.

Motivated by these examples, we systematically explored the network-based separation of each tissue pair, measuring the shortest network-based separations $d_{AB}$ between gene sets expressed at $z_E \geqslant 1.0$. We find that of the 2,016 possible pairs, for 1,415 the network-based separation is significantly different from the corresponding random expectations. Of these, 851 disease pairs are closer than expected by chance, while 564 tissue pairs show a statistically significant separation from each other.

In summary, we created a list of significant inter-tissue $d_{AB}$ separations, which we provide in text format.

### C. The Hierarchy of Tissue-Tissue Inter-Relationships

The obtained 1,415 significant network-based separations between genes expressed in different tissues allow us to build a dendrogram for the hierarchy of tissue clusters, using Ward's method [21] The dendrogram is depicted in Fig. S5A. We color tissues according to their type.

The dendrogram predicts three major clusters, a cluster of reproductive organ tissues (yellow), a cluster of immune system cells (green), and a cluster of nervous system tissues, which consists of several subclusters (blue). The corresponding clustering heat map is shown in Fig. S5B.

## V. DISEASE-TISSUE BIPARTITE NETWORK

In order to analyze all disease-tissue manifestations systematically, we identified all diseases that form a statistically significant connected component in a particular tissue. To test if disease $d$ has a significant connected component in tissue $t$, we first compile the tissue-specific interactome that consists of genes with expression significance $z_E \geqslant 1.0$ in tissue $t$ and interactions between them. Next, we map genes associated with disease $d$ onto the tissue-specific interactome and then measure the size of the largest connected component $S$ and the total number of disease genes $S_{total}$ expressed in tissue $t$ (Fig. 4A).

To test the significance of the observed disease module we assume (null hypothesis) that disease genes do not preferentially interact in the tissue-specific interactome. With this null hypothesis, we select $S_{total}$ genes randomly in the tissue-specific interactome and determine the resulting size of the largest connected component $S^{rand}$. We repeat the same procedure 1,000 times to obtain the distribution $P^{rand}(S)$. Assuming the normality of $P^{rand}(S)$, we use the $z$-score to compute the significance of the real data

$$z = \frac{S - \langle S^{rand} \rangle}{\sigma(S^{rand})}, \tag{22}$$

where $\langle S^{rand} \rangle$ and $\sigma(S^{rand})$ are the mean and the standard deviations of $P^{rand}(S)$. Assuming the normality of $P^{rand}(S)$, we use the threshold of $z \geqslant 1.6$ for modules to be larger than expected by chance with significance $p$-value$\leqslant 0.05$.

This strategy allows us to analyze systematically all disease-tissue pairs and to build a disease-tissue bipartite network that links diseases to tissues if the observed tissue-specific disease module has statistically significant size. The resulting bipartite network links 70 diseases to 64 tissues via 187 links (Fig. S6). While some diseases manifest in as many as 12 tissues, on average, each of the diseases included in the network manifest in 2.4 tissues. Tissues with the largest number of expressed diseases are BDCA4 dentritic cells (18 diseases), X721 B lymphoblast cells (16), and CD56 NKcells (12), appearing as the hubs of the disease-tissue network. For 35 diseases, we did not find a statistically significant module in any tissue, and 29 tissues did not have any disease associated with them, among the 70 diseases included in this analysis. Plausible reasons for these latter two findings are the incompleteness of the interactome and the limited number of known disease genes. The resulting bipartite network is provided in text format as a separate file.

### A. Robustness of the Disease-Tissue Bipartite Network

To test the robustness of the disease-tissue bipartite network with respect to the expression significance threshold, we the constructed disease-tissue bipartite network for a more stringent threshold of $z_E^* = 2.0$. The bipartite disease-tissue network obtained form $z_E^* = 2.0$ is an almost exact subset of the original network obtained for $z_E^* = 1.0$. Indeed, the resulting network has a total of $E = 76$ links; 73 of the 76 links coincide with those of the original network calculated at $z_E = 1.0$. Three disease-tissue links contained in the network obtained for $z_E^* = 2.0$ and not in the original network obtained for $z_E^* = 2.0$ are: carbohydrate metabolism, inborn errors and CD34 ($z = 2.15$); nutritional and metabolic diseases and amygdala ($z = 2.56$); and the pair of nutritional and metabolic diseases and thalamus ($z = 1.78$). We also note that statistical significance values of links mutually observed in both networks are strongly correlated (Pearson $r = 0.81$, Fig. S7).

## VI. TISSUE-SPECIFIC FILTERING OF DISEASE GENES.

Here we explore the effects of tissue-specificity on genes associated with arthritis. Arthritis is a form of joint disorder that involves inflammation in one or more joints. Arthritis is characterized by synovial lining hyperplasia and chronic infiltration by T and B cell and dendritic cells (DC). It has been observed that the frequencies of BDCA4+ DC in peripheral blood of arthritis patients are decreased compared with healthy controls and non-arthritis patients [22].

Figure. S8A depicts the correlation between the significance of GWAS associations and gene expression levels in BDCA4 dentritic cells for arthritis. All 31 genes expressed at $z_E < 1.0$, are characterized by relatively low GWAS significance ($-\text{Log}\,(p) < 100$). Of the 21 genes expressed at $z_E \geqslant 1.0$, 5 have GWAS significance of ($-\text{Log}\,(p) > 200$) and 16 have GWAS significance of ($12 < -\text{Log}\,(p) < 250$) (Fig . S8A). To analyze interactions among expressed genes we construct tissue-specific subnetwork of arthritis genes (Fig. S8B). We find that of 5 genes with GWAS significance $-\text{Log}\,(p) > 200$ ($HLA-DRB1$, $HLA-DRB5$, $HLA-DQA1$, $HLA-DQA2$, and $HLA-DQB1$), 3 genes ($HLA-DQA1$, $HLA-DQA2$, and $HLA-DQB1$) form a connected component in BDCA4 dentritic cells.

The largest rheumatoid arthritis genetic susceptibility effect is conferred by the $HLA$ locus and studies conducted earlier identified multiple RA risk alleles within the $HLA-DRB1$ gene [23]. A recent study has shown a SNP in $HLA-DQA1$ associated with RA both in UK Caucasian populations and North American [24]. It has also been proposed that $HLA-DQB1$ polymorphism determines incidence, onset, and severity of collagen-induced arthritis in transgenic mice [25]. Markers in genes $HLADRA$, $HLADRB5$, $HLADRB1$, $HLADQA1$, $HLADQB1$, $HLADQA2$, and $TAP2$ were among the most significantly associated markers ($p < 10^{-10}$) in case control analysis of 855 RA patients and 977 controls [26].

Thus, tissue specific arthritis module in BDCA4 dentritic cells can serve as another example of an effective filter, aggregating disease genes with high GWAS significance and keeping less relevant genes apart from disease module. We note that even though $HLA-DRB1$ and $HLADRB5$ have high GWAS significance, these genes are not part of the arthritis module. A possible reason could be the incompleteness of the human interactome.

## VII. FUNCTIONAL SIMILARITY IS A NECESSARY CONDITION FOR DISEASE TISSUE-SPECIFICITY

Our analysis of tissue-specific disease modules in the main text indicates that disease genes expressed in a tissue in which a given disease has a significant connected component are functionally more uniform. This result implies that disease manifestation in a particular tissue is related to the similarity between functions or perturbations of functions performed by disease genes and functions performed by that tissue.

To test this hypothesis we inferred sets of GO functional characteristics of all diseases and tissues in our dataset. Specifically, for every tissue $t$ in our dataset we inferred its set of functions $F_t$ by performing GO enrichment analysis of expressed genes [27, 28]. Similarly, for every disease $d$ in our dataset we inferred its set of functions $F_d$ using GO enrichment of genes associated with this disease. As a result, we characterized every disease and every tissue in our dataset by a characteristic set of enriched functions. To assess the functional similarity of a particular disease-tissue pair we computed the Simpson similarity coefficient $S(F_d, F_t)$ of the corresponding sets of enriched functions. We repeated this calculation for every disease tissue pair and identified 3017 (of total 4480) disease tissue pairs with non-zero functional similarity.

We found that all 187 disease-tissue specificity associations established in our work are among the 3017 disease tissue pairs with non-zero functional overlap. To check if one could identify disease-tissue specificity associations by solely relying on disease-tissue functional similarities we calculated the Receiver Operating Characteristic (ROC) curve by comparing disease-tissue specificity pairs with functionally similar disease-tissue pairs (Fig. S9). While the

ROC curve lies significantly above the diagonal, the predictive power of disease-tissue similarity metric is far from perfect, yielding the Area Under the Curve (AUC) statistic, $AUC = 0.72$.

Taken together, our results suggest that disease-tissue functional similarity is a necessary but not sufficient criterium for disease manifestation in the tissue. Indeed, in order to manifest in the specific tissue disease must perturb some of its functions. At the same time, significant functional similarity of a given disease-tissue pair does not necessarily improve chances of disease manifestation, as it might be sufficient for a disease to perturb only one or several tissue functions.

---


[1] Matys V, Fricke E, Geffers R, Gößling E, Haubrock M, Hehl R, et al. TRANSFAC®: transcriptional regulation, from patterns to profiles. Nucleic acids research. 2003;31(1):374–378.

[2] Stelzl U, Worm U, Lalowski M, Haenig C, Brembeck FH, Goehler H, et al. A human protein-protein interaction network: a resource for annotating the proteome. Cell. 2005;122(6):957–968.

[3] Yu H, Tardivo L, Tam S, Weiner E, Gebreab F, Fan C, et al. Next-generation sequencing to generate interactome datasets. Nature methods. 2011;8(6):478–480.

[4] Venkatesan K, Rual JF, Vazquez A, Stelzl U, Lemmens I, Hirozane-Kishikawa T, et al. An empirical framework for binary interactome mapping. Nature methods. 2009;6(1):83–90.

[5] Rual JF, Venkatesan K, Hao T, Hirozane-Kishikawa T, Dricot A, Li N, et al. Towards a proteome-scale map of the human protein–protein interaction network. Nature. 2005;437(7062):1173–1178.

[6] Rolland T, Taşan M, Charloteaux B, Pevzner SJ, Zhong Q, Sahni N, et al. A Proteome-scale map of the human interactome network. Cell. 2014;159(5):1212–1226.

[7] Aranda B, Achuthan P, Alam-Faruque Y, Armean I, Bridge A, Derow C, et al. The IntAct molecular interaction database in 2010. Nucleic acids research. 2010;38(suppl 1):D525–D531.

[8] Ceol A, Aryamontri AC, Licata L, Peluso D, Briganti L, Perfetto L, et al. MINT, the molecular interaction database: 2009 update. Nucleic acids research. 2009;p. gkp983.

[9] Stark C, Breitkreutz BJ, Chatr-Aryamontri A, Boucher L, Oughtred R, Livstone MS, et al. The BioGRID interaction database: 2011 update. Nucleic acids research. 2011;39(suppl 1):D698–D704.

[10] Prasad TK, Goel R, Kandasamy K, Keerthikumar S, Kumar S, Mathivanan S, et al. Human protein reference database2009 update. Nucleic acids research. 2009;37(suppl 1):D767–D772.

[11] Lee DS, Park J, Kay K, Christakis N, Oltvai Z, Barabási AL. The implications of human metabolic network topology for disease comorbidity. Proceedings of the National Academy of Sciences. 2008;105(29):9880–9885.

[12] Ruepp A, Waegele B, Lechner M, Brauner B, Dunger-Kaltenbach I, Fobo G, et al. CORUM: the comprehensive resource of mammalian protein complexes2009. Nucleic acids research. 2009;p. gkp914.

[13] Hornbeck PV, Kornhauser JM, Tkachev S, Zhang B, Skrzypek E, Murray B, et al. PhosphoSitePlus: a comprehensive resource for investigating the structure and function of experimentally determined post-translational modifications in man and mouse. Nucleic acids research. 2011;p. gkr1122.

[14] Hamosh A, Scott AF, Amberger JS, Bocchini CA, McKusick VA. Online Mendelian Inheritance in Man (OMIM), a knowledgebase of human genes and genetic disorders. Nucleic acids research. 2005;33(suppl 1):D514–D517.

[15] Mottaz A, Yip YL, Ruch P, Veuthey AL. Mapping proteins to disease terminologies: from UniProt to MeSH. BMC bioinformatics. 2008;9(Suppl 5):S3.

[16] Ramos EM, Hoffman D, Junkins HA, Maglott D, Phan L, Sherry ST, et al. Phenotype–Genotype Integrator (PheGenI): synthesizing genome-wide association study (GWAS) data with existing genomic resources. European Journal of Human Genetics. 2014;22(1):144–147.

[17] Su AI, Wiltshire T, Batalov S, Lapp H, Ching KA, Block D, et al. A gene atlas of the mouse and human protein-encoding transcriptomes. Proceedings of the National Academy of Sciences of the United States of America. 2004;101(16):6062–6067.

[18] Ashburner M, Ball CA, Blake JA, Botstein D, Butler H, Cherry JM, et al. Gene Ontology: tool for the unification of biology. Nature genetics. 2000;25(1):25–29.

[19] Berriz GF, Beaver JE, Cenik C, Tasan M, Roth FP. Next generation software for functional trend analysis. Bioinformatics. 2009;25(22):3043–3044.

[20] Menche J, Sharma A, Kitsak M, Ghiassian SD, Vidal M, Loscalzo J, et al. Uncovering disease-disease relationships through the incomplete interactome. Science. 2015;347(6224). Available from: `http://www.sciencemag.org/content/347/6224/1257601.abstract`.

[21] Ward Jr JH. Hierarchical grouping to optimize an objective function. Journal of the American statistical association. 1963;58(301):236–244.

[22] Lebre M, Tas S, Reinders-Blankert P, Smeets T, Tak P. Characterization of BDCA1 and BDCA4 dendritic cell subsets in rheumatoid arthritis patients. Arthritis research & therapy. 2004;6(Suppl 1):46.

[23] Viatte S, Plant D, Raychaudhuri S. Genetics and epigenetics of rheumatoid arthritis. Nature Reviews Rheumatology. 2013;9(3):141–153.

[24] Eleftherohorinou H, Hoggart CJ, Wright VJ, Levin M, Coin LJ. Pathway-driven gene stability selection of two rheumatoid arthritis GWAS identifies and validates new susceptibility genes in receptor mediated signalling pathways. Human molecular genetics. 2011;20(17):3494–3506.

[25] Bradley DS, Nabozny GH, Cheng S, Zhou P, Griffiths MM, Luthra HS, et al. HLA-DQB1 polymorphism determines incidence, onset, and severity of collagen-induced arthritis in transgenic mice. Implications in human rheumatoid arthritis. Journal of Clinical Investigation. 1997;100(9):2227.

[26] Vignal C, Bansal AT, Balding DJ, Binks MH, Dickson MC, Montgomery DS, et al. Genetic association of the major histocompatibility complex with rheumatoid arthritis implicates two non-DRB1 loci. Arthritis & Rheumatism. 2009;60(1):53–62.

[27] Berriz GF, King OD, Bryant B, Sander C, Roth FP. Characterizing gene sets with FuncAssociate. Bioinformatics. 2003;19(18):2502–2504.

[28] Guney E, Menche J, Vidal M, Barábasi AL. Network-based in silico drug efficacy screening. Nature communications. 2016;7.

## VIII. SUPPLEMENTARY FIGURES

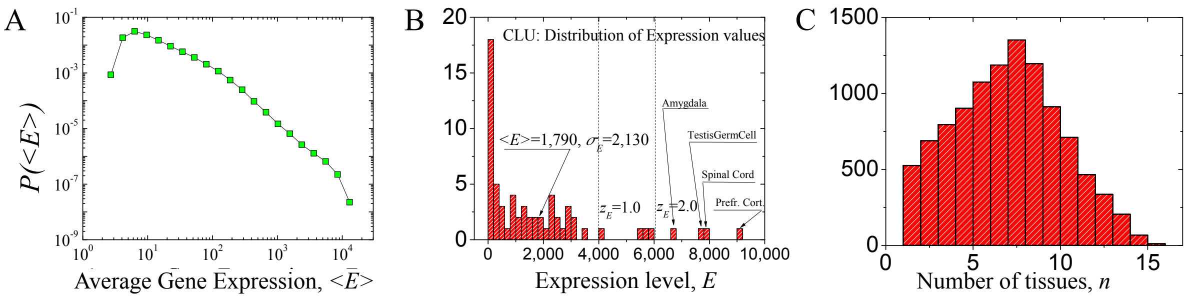


FIG. S1. Gene expression significance. (A) For each gene $i$ we calculate its average expression $\langle E(i) \rangle$ across all considered tissues. The figure depicts the distribution of the obtained $\langle E_i \rangle$ values. Data is binned logarithmically. (B) The distribution of expression values for the clusterin ($CLU$). The two vertical lines correspond to significance threshold $z_E^* = 1.0$ and $z_E^* = 2.0$ respectively. (C) Tissue specificity of individual genes. The histogram depicts the count of genes that are simultaneously expressed in exactly $n$ tissues. On average each gene is expressed in 6.5 tissues.

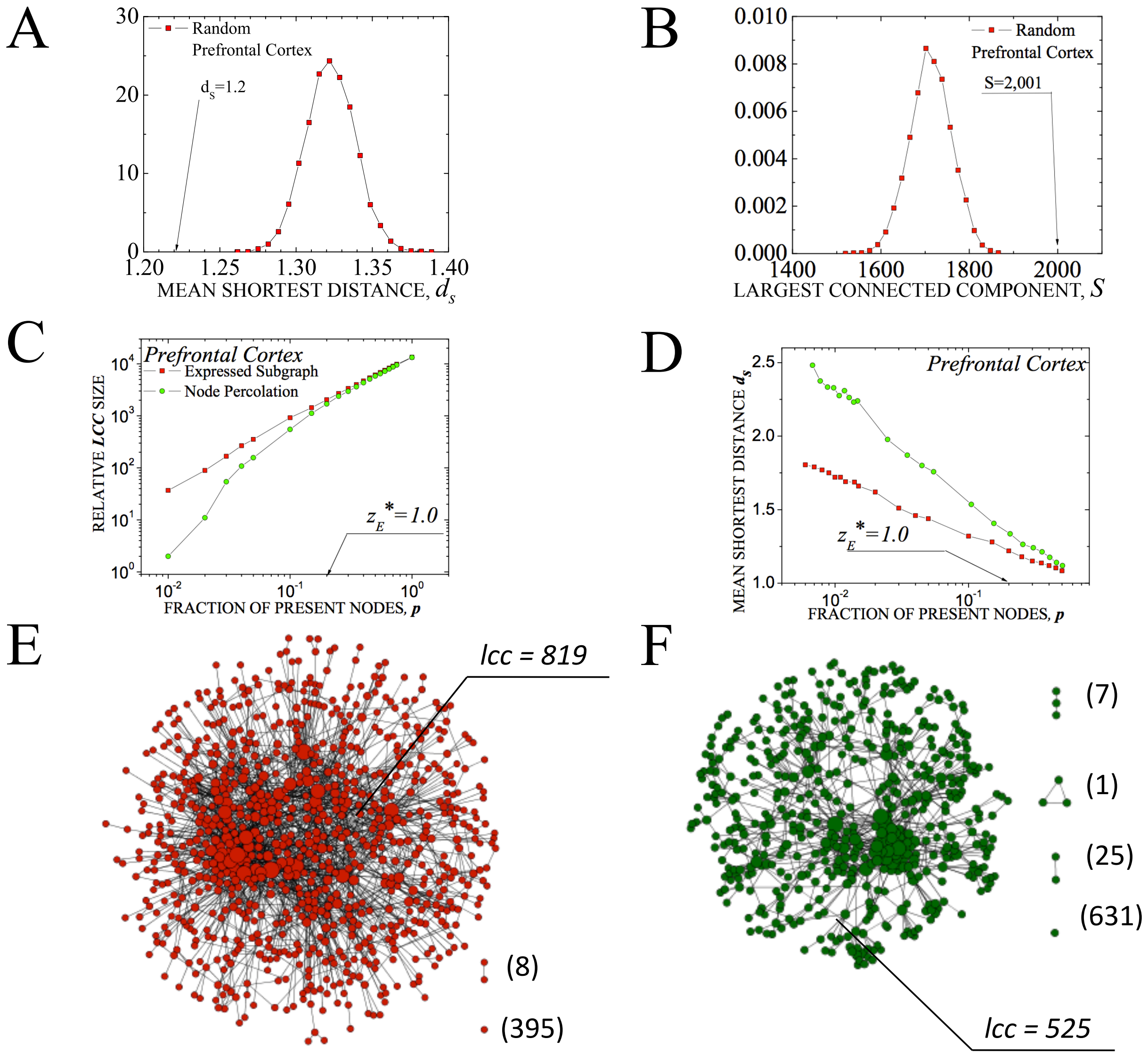


FIG. S2. Network-based localization of genes expressed in prefrontal cortex. (A) The distribution of the mean shortest network-based separation $d_S$ for randomly chosen sets of genes in the human interactome. (B) The distribution of connected components size, $S$, for randomly chosen sets of genes in the human interactome. (C) Relative size of the largest connected component (lcc) of genes expressed in the prefrontal cortex as a function of the fraction $p$ of present nodes. Nodes are added in the decreasing order of expression significance. (D) The mean shortest distance $d_S$ between genes expressed in the prefrontal cortex as a function of the fraction $p$ of present nodes. Nodes are added in the decreasing order of expression significance. (E) The subgraph of 1,230 genes expressed in the prefrontal cortex with significance $z_E \leqslant 2.0$. (F) The subgraph of randomly chosen 1,230 genes. The number of genes in the random subgraph is chosen to match the number of genes expressed in the prefrontal cortex at $z_E \leqslant 2.0$.

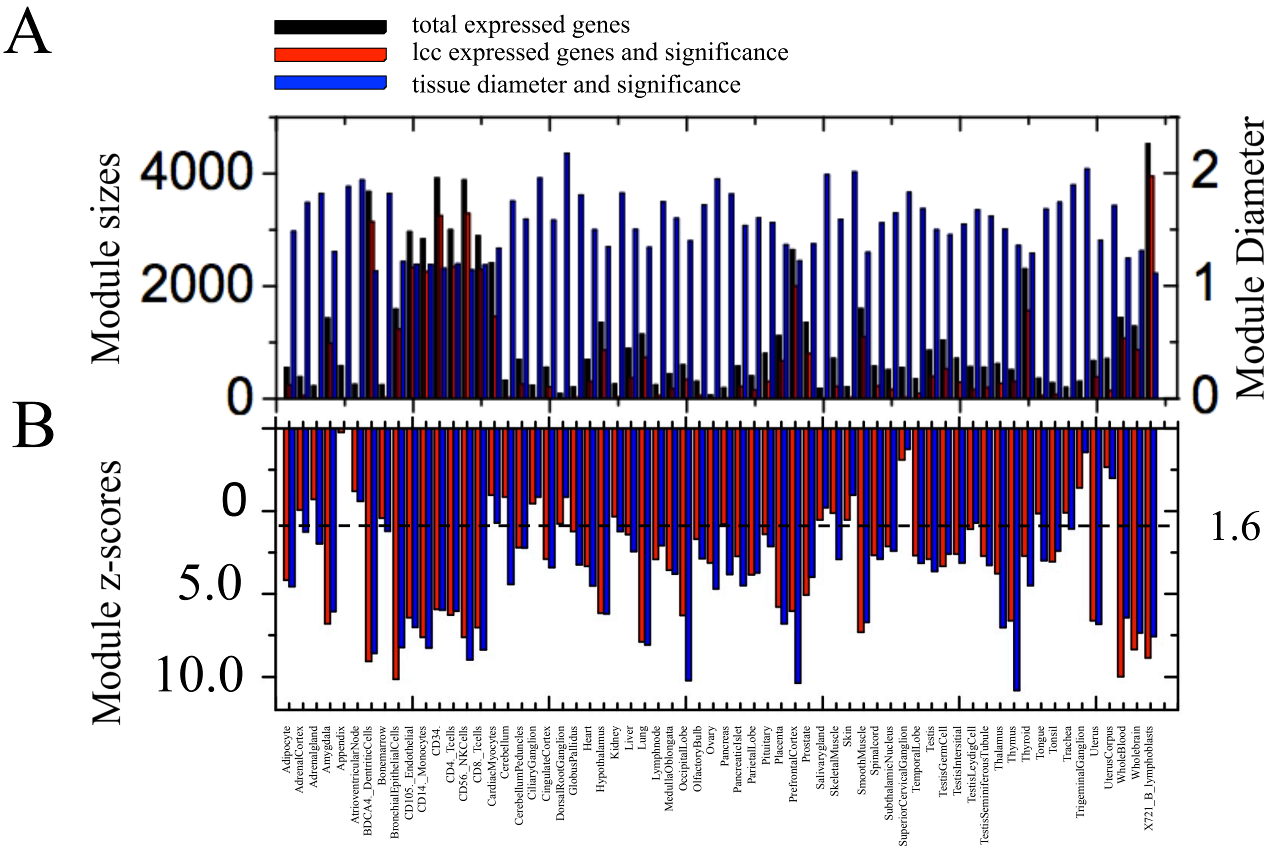


FIG. S3. Localization statistics for 64 tissues. (A) The total number of expressed genes (black), the number of genes constituting the largest connected component (module size, red), and the mean shortest separation, $d_S$ (module diameter, blue), calculated for considered 64 tissues. (B) The significance of the observed largest connected components (red) and the significance of the mean shortest separation (blue). The horizontal dashed line corresponds to $z = 1.6$.

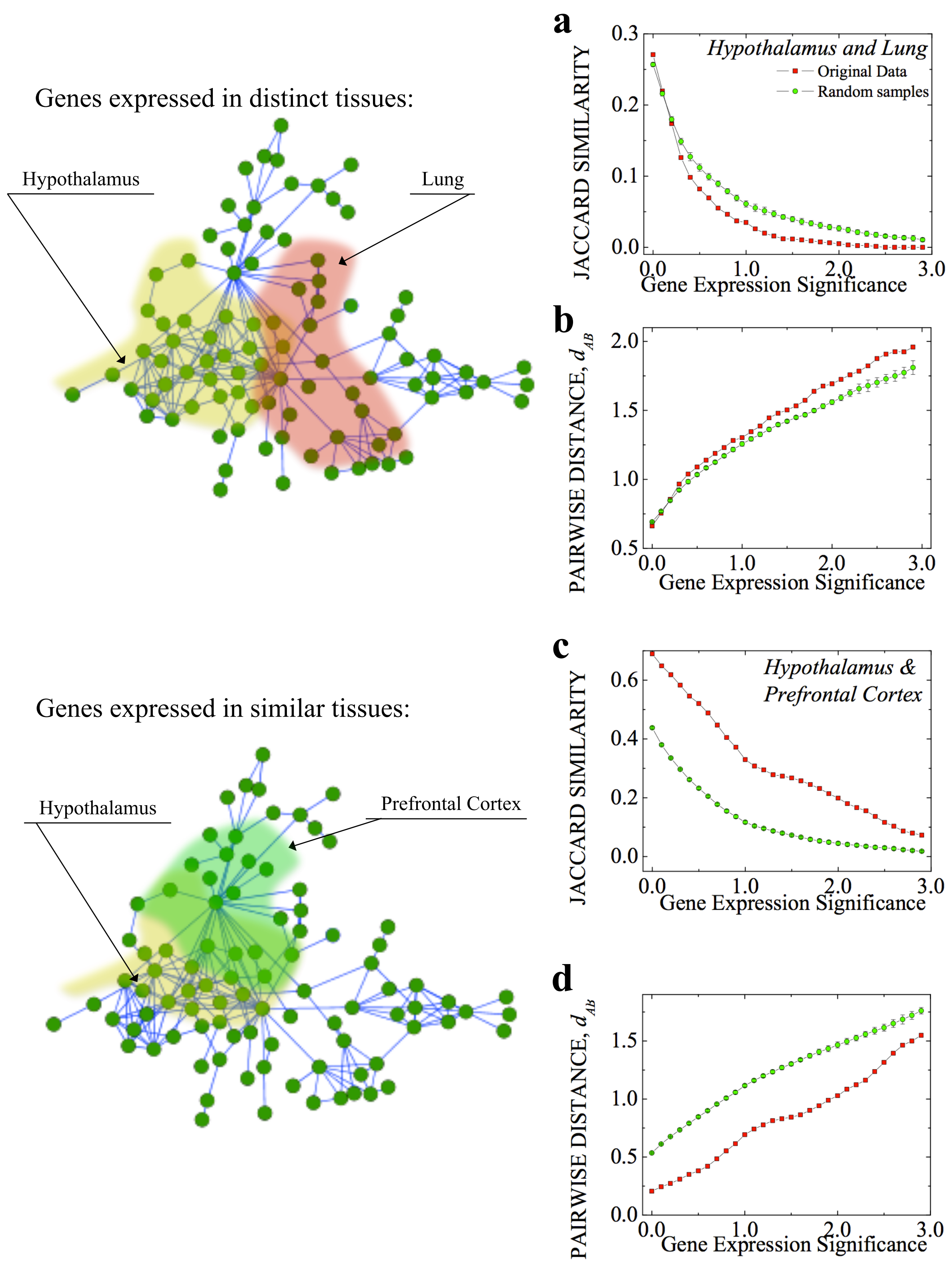


FIG. S4. Genes expressed in different tissues. (A) The Jaccard index and (B) the mean shortest separation $d_{AB}$ calculated for sets of genes expressed in the hypothalamus and the lung. (C) The Jaccard index and (D) the mean shortest separation $d_{AB}$ calculated for sets of genes expressed in the hypothalamus and the prefrontal cortex. Red points correspond to original data, while green points correspond to random samples.

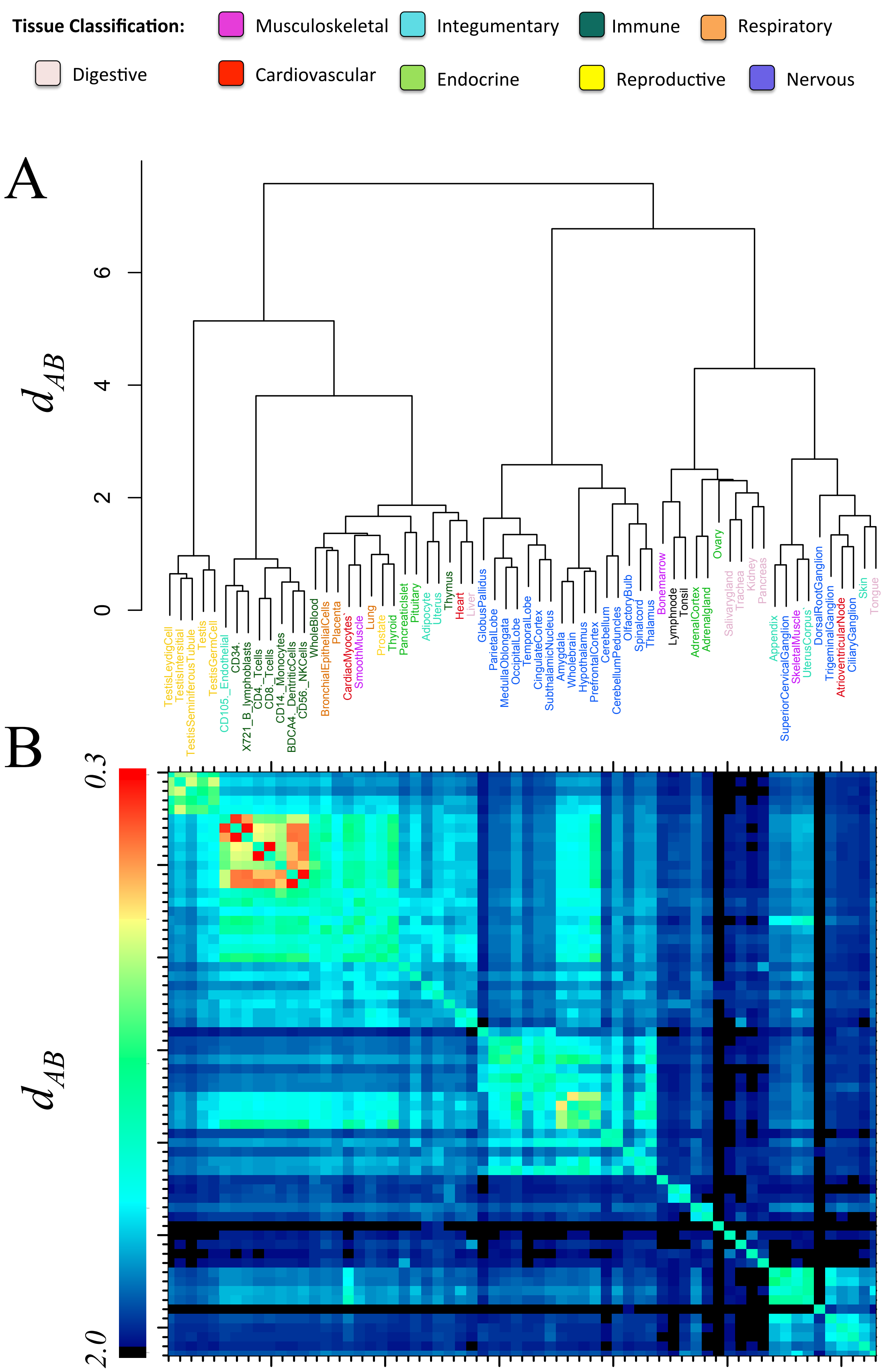


FIG. S5. Ward's hierarchical clustering of 64 tissues.

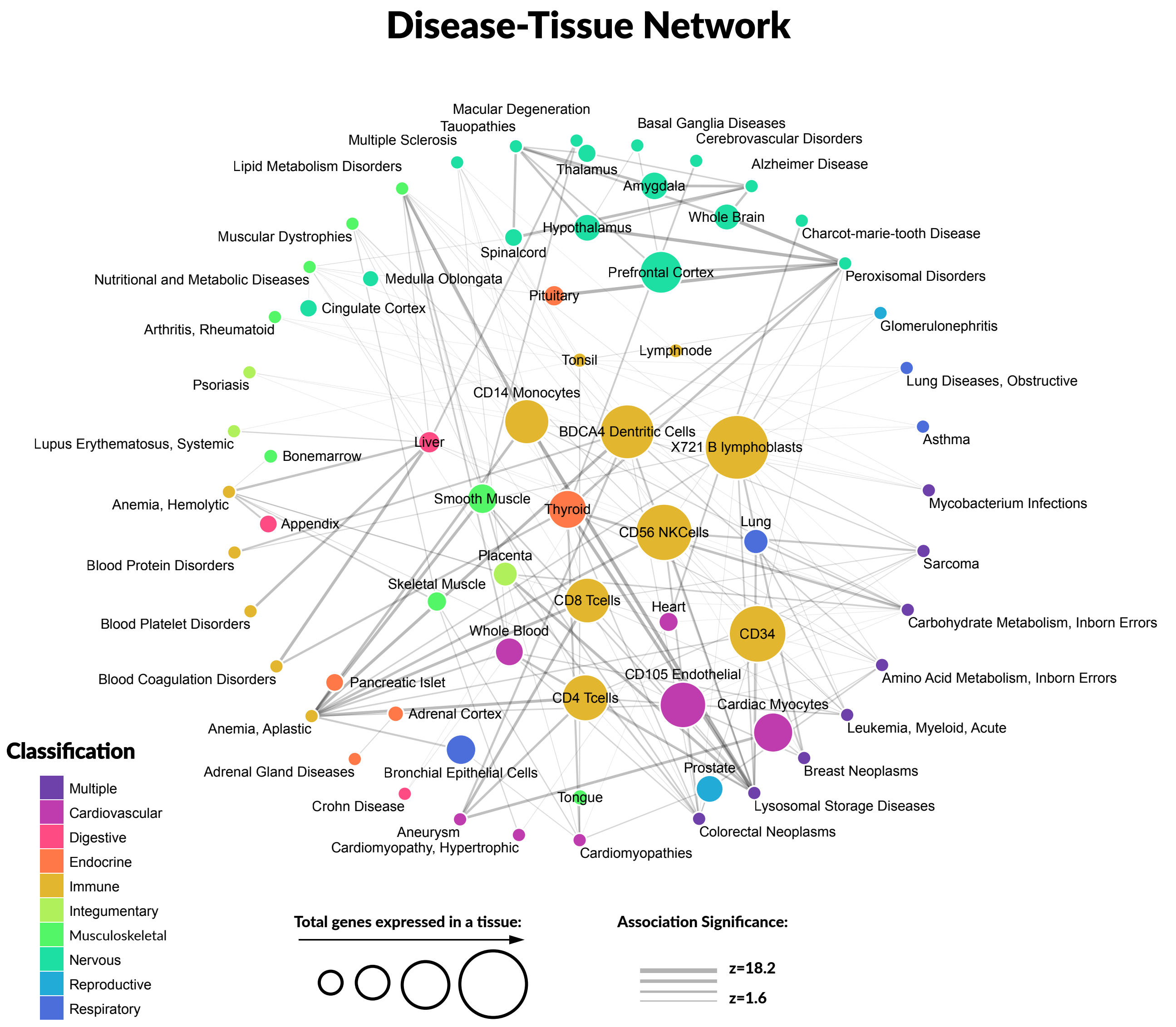


FIG. S6. Disease-tissue bipartite network. Tissues are placed within the circle while disease are positioned along the circumference. Nodes are colored according to tissue classification. The sizes of tissue nodes are proportional to the total number of genes expressed in them. The widths of connecting links correspond to the significance of the association.

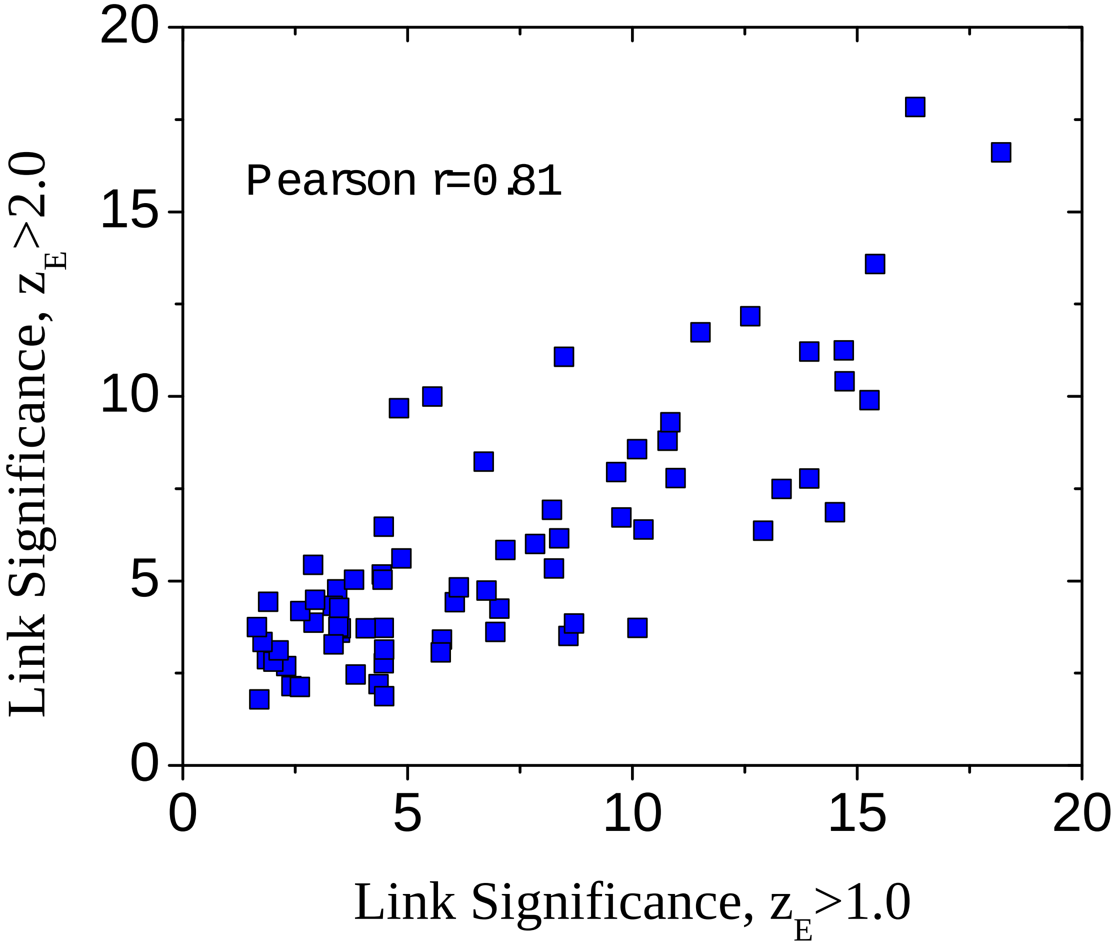


FIG. S7. The robustness of the disease-tissue bipartite network. $z$-score significance of disease-tissue links in the bipartite network obtained for $z_E^* = 2.0$ as a function of the $z$-score significance of the same links in the bipartite network obtained for $z_E^* = 1.0$.

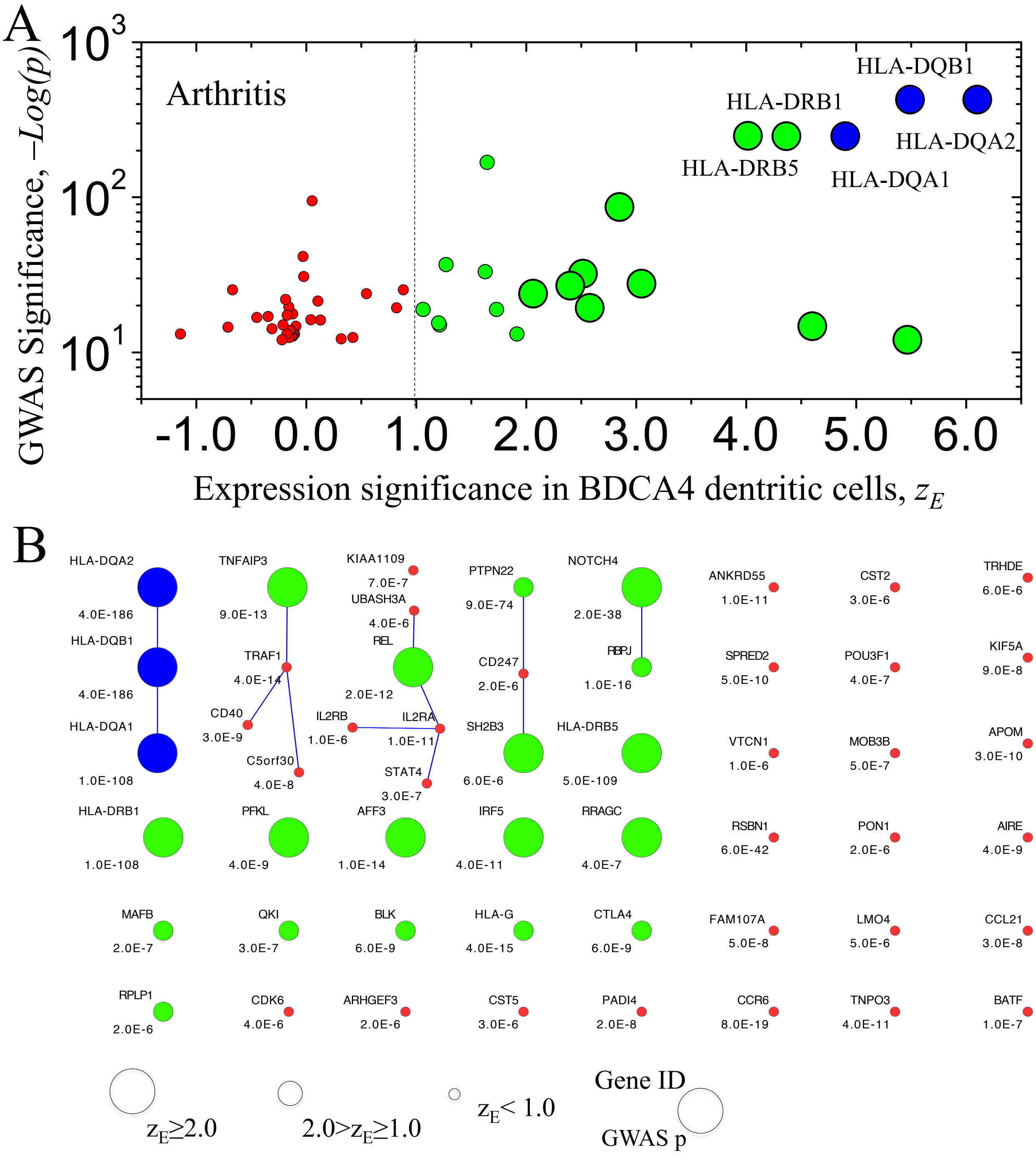


FIG. S8. **Tissue-specific filtering: arthritis.** To demonstrate the filtering effect of tissue-specificity we consider genes associated with arthritis. Node sizes correspond to gene expression significance in BDCA4 dentritic cells. Blue nodes correspond to genes in the connected component of arthritis with expression significance of $z_E > 1.0$. Green nodes correspond to genes with $z_E > 1.0$ which are not part of the connected component. Red nodes correspond to genes with $z_E < 1.0$. (A) The scatter plot of GWAS association significance as a function of gene expression significance in BDCA4 dentritic cells. (B) Arthritis-associated genes and links among them. Numeric values correspond to GWAS association significance. The most relevant arthritis genes form the connected component.

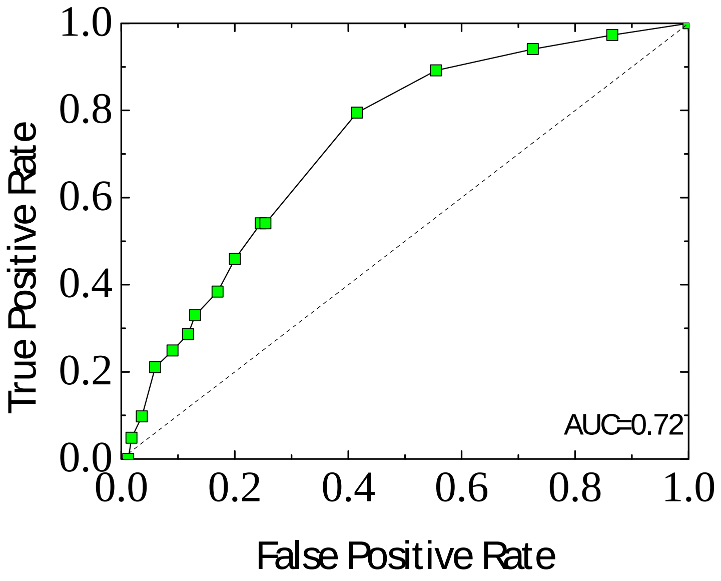


FIG. S9. The Receiver Operating Characteristic (ROC) curve computed for the comparison of disease-tissue specific associations and disease-tissue similar pairs. To calculate the ROC curve we ranked disease-tissue pairs by their functional similarity. By varying the fraction of present most similar disease-tissue pairs we measured the fraction of correctly identified disease-tissue specificity pairs (True Positive Rate) and the fraction of misidentified disease-tissue specificity pairs (False Positive Rate).
