# Supplementary material for: Tissue Specificity of Human Disease Module
Source: Sci Rep. 2016 Oct 17;6:35241. doi: 10.1038/srep35241 (PMC5066219; doi:10.1038/srep35241)
Supplement: Supplementary Data File [file srep35241-s2.docx]

“Tissue Specificity of Human Disease Module” by Maksim Kitsak, Amitabh Sharma, Joerg Menche, Emre Guney, Susan Dina Ghiassian, Joseph Loscalzo & Albert-László Barabási.

We provide following supplementary datasets:

1. interactome.tds (Table containing the human interactome),

2. disease_tissue_pairs_data.tds (File containing all disease tissue pairs with expression significance),

3. gene_tissue_expression_significance.tds (Gene expression significance data).
